# The effect of dual task-oriented circuit training on neuroplasticity and walking adaptability in subacute stroke patients: A pilot and feasibility study

**DOI:** 10.1371/journal.pone.0361255

**Published:** 2026-10-27

**Authors:** Farida Arisanti, Bejo Ropii, Saniy Ghaida, Tsuchiya Tristiana Dewi, Bernadeta Cahyo Setianingrum Poernomo, Yunia Sribudiani, Lisda Amalia, Julia Patrick Engkasan, Vitriana Biben

**Affiliations:** 1 Faculty of Medicine, Doctoral Study Program in Medical Science, Universitas Padjadjaran, Kabupaten Sumedang, Indonesia; 2 Department of Physical and Rehabilitation Medicine, Faculty of Medicine Universitas Padjadjaran, Kabupaten Sumedang, Indonesia; 3 Preanger Institute for Biomedical Engineering, Science, and Technology, Bandung, Indonesia; 4 Kabupaten Sumedang, Physical Medicine and Rehabilitation Study Program, Faculty of Medicine, Universitas Padjadjdaran, Kabupaten Sumedang, Indonesia; 5 Dustira Hospital, Bandung, Indonesia; 6 Department of Biomedical Sciences, Faculty of Medicine, Universitas Padjadjaran, Kabupaten Sumedang, Indonesia; 7 Department of Neurology, Faculty of Medicine, Universitas Padjadjaran, Kabupaten Sumedang, Indonesia; 8 Department of Rehabilitation Medicine, Faculty of Medicine, Universiti Malaya, Kuala Lumpur, Malaysia; Nathan S Kline Institute, UNITED STATES OF AMERICA

## Abstract

**Background:**

Stroke remains a leading cause of long-term disability worldwide, with a substantial proportion of survivors experiencing persistent limitations in functional mobility. Although many individuals regain the ability to walk in structured clinical environments, only a minority achieve independent community ambulation due to cognitive–motor interference (CMI). The subacute phase is characterized by heightened neuroplastic potential and responsiveness to rehabilitation. Dual Task-Oriented Circuit Training (DTOCT) integrates task-oriented circuit training with concurrent cognitive challenges and may provide a functionally relevant approach to addressing cognitive–motor interference. However, its feasibility, safety, and preliminary effects in subacute stroke remain unclear.

**Methods:**

This three-arm, participant-blinded, randomized pilot and feasibility trial will recruit 36 individuals with subacute stroke and allocate them equally to DTOCT, Task-Oriented Circuit Training (TOCT), or Dual Task Training (DTT). Participants will receive three supervised sessions per week for 4 weeks (12 sessions). Primary outcomes will assess feasibility and safety, including adherence, retention, adverse events, heart rate, blood pressure, and perceived exertion. Secondary exploratory outcomes will include walking endurance assessed using the 6-Minute Walk Test, gait speed using the 10-Meter Walk Test, walking adaptability using the Standardized Walking Obstacle Course, and serum brain-derived neurotrophic factor as an exploratory peripheral biomarker associated with neuroplasticity.

**Discussion:**

This pilot study is designed to determine the feasibility and safety of DTOCT and to generate preliminary effect estimates for clinical and neurobiological outcomes. The findings will inform primary outcome selection, intervention refinement, and sample-size estimation for a subsequent adequately powered randomized controlled trial rather than establish definitive efficacy or superiority.

**Trial registration:**

ClinicalTrials.gov. Identifier: NCT07494357 (registered prospectively). Protocol ID: IKFR-202603.01)

## 1. Introduction

Stroke remains one of the leading causes of long-term disability worldwide, imposing a substantial burden on individuals [1]. Despite advances in acute management, a large proportion of stroke survivors experience persistent impairments in motor, cognitive, and functional domains, particularly in mobility and walking ability [2]. While many patients regain the capacity to walk in structured clinical environments, a significant gap persists between clinical recovery and real-world functional independence. It has been reported that although up to 80% of stroke survivors achieve independent walking in controlled settings, only approximately 30% are able to safely navigate complex community environments that require simultaneous cognitive and motor processing [3,4]. This discrepancy is largely attributed to CMI, defined as a decline in motor performance, cognitive performance, or both when motor and cognitive tasks are performed simultaneously compared with performance under single-task conditions [5]. In healthy individuals, walking is predominantly an automatic process mediated by subcortical neural circuits [6]. Following stroke, however, damage to motor and associative pathways may increase reliance on conscious attentional and executive control during gait [7]. When attentional resources are insufficient to meet the combined demands of walking and a concurrent cognitive task, gait speed, stability, obstacle negotiation, or cognitive performance may deteriorate. This is particularly relevant to community ambulation, where individuals are frequently required to divide attention while navigating obstacles, changing direction, monitoring traffic, communicating, or carrying objects. Consequently, CMI may contribute to reduced walking safety, increased fall risk, and limited independence in real-world environments.

The subacute phase of stroke (2 weeks to 5 months post-onset) is characterized by heightened potential for neuroplastic change, including synaptic reorganization, changes in cortical excitability, and increased responsiveness to rehabilitation [8]. Although often described as a “golden window” for recovery, neuroplasticity is not restricted to this phase and may continue during later stages after stroke. Nevertheless, the subacute period remains clinically relevant because rehabilitation may interact with ongoing spontaneous and experience-dependent neural reorganization, with recovery potentially influenced by the intensity, specificity, and complexity of therapeutic interventions. Traditional rehabilitation approaches, including Task-Oriented Training and its circuit-based form, TOCT, have demonstrated effectiveness in improving gait speed, endurance, and functional mobility through repetitive, goal-directed motor practice [9–13]. However, these approaches are often performed in controlled environments with minimal cognitive demand, limiting their ecological validity and transferability to real-world settings.

To address these limitations, DTT has been introduced to specifically target cognitive–motor integration by requiring patients to perform cognitive tasks concurrently with motor activities [4]. Although DTT may improve dual-task performance and attentional control during gait, it is commonly delivered in relatively simplified walking-based formats. In contrast, TOCT emphasizes repetitive, goal-directed functional motor practice performed across multiple task-oriented stations, primarily targeting physical capacity, gait, balance, and functional mobility. Combining these approaches is particularly relevant because cognitive–motor interference can compromise gait stability during dual-task walking after stroke [14]. DTOCT therefore embeds concurrent cognitive demands within progressively challenging task-oriented circuit activities, simultaneously targeting motor performance, cognitive processing, and functional adaptability. The expected added therapeutic value of DTOCT lies not simply in greater task complexity, but in the opportunity to train cognitive–motor integration while maintaining the repetitive, functionally relevant, and physically demanding characteristics of circuit-based rehabilitation. This integrated approach may engage complementary neuroplastic mechanisms, whereby aerobic and functional motor activity promotes systemic neurotrophic responses while cognitive engagement increases demands on higher-order cortical networks. In particular, BDNF plays a central role in synaptic plasticity, motor learning, and neuronal survival, and has been investigated as a peripheral biomarker associated with rehabilitation-related neuroplastic responses [15,16]. Complementary evidence from chronic stroke also suggests that combining proprioceptive or postural training with dual-task exercises may improve gait-related outcomes, further supporting the integration of cognitive, motor, and postural demands within functionally oriented rehabilitation [17].

Despite its theoretical rationale, evidence on DTOCT remains limited, particularly during the subacute phase of stroke recovery. Previous studies have largely examined motor and cognitive training separately, with limited investigation of integrated approaches that reflect real-world mobility demands or combine functional walking outcomes with neurobiological measures such as serum BDNF. Accordingly, the primary research question of this pilot study is whether DTOCT can be delivered feasibly and safely in individuals with subacute stroke, as reflected by adherence, retention, adverse events, and physiological tolerability. Secondary exploratory research questions examine whether DTOCT is associated with potentially meaningful changes in walking endurance, gait speed, walking adaptability, and serum BDNF compared with TOCT and DTT. This pilot and feasibility trial is intended to generate preliminary effect estimates and information on intervention implementation rather than to establish definitive efficacy. The findings will inform the design, outcome selection, and sample-size estimation of a subsequent adequately powered randomized controlled trial.

## 2. Methods

### 2.1. Study design

This study is designed as a three-arm, parallel-group, participant-blinded, randomized pilot and feasibility trial registered at ClinicalTrials.gov (Identifier: NCT07494357; Protocol ID: IKFR-202603.01) to evaluate the effects of DTOCT in individuals with subacute stroke. Participants will be allocated into three intervention groups:

a) DTOCT
b) TOCT
c) DTT

All groups will receive supervised rehabilitation interventions over a 4-week period, with outcome assessments conducted at baseline and post-intervention. Patients and members of the public were not involved in the design, conduct, reporting, or dissemination planning of this pilot study. The intervention protocol was developed by rehabilitation physicians and researchers based on current evidence and clinical expertise. Participant feedback regarding the feasibility and acceptability of the intervention will be collected during the study and used to refine the design of a future definitive randomized controlled trial.

### 2.2. Study setting

The study will be conducted at RS TK.II 03.05.01 Dustira, Bandung, Indonesia, a tertiary healthcare facility providing specialized neurorehabilitation services. All intervention sessions and outcome assessments will take place within the rehabilitation unit under the supervision of trained physiatrists and rehabilitation therapists.

### 2.3. Participants

Participants will be screened based on the following criteria

Inclusion Criteria

a) Diagnosis of first-ever subacute stroke, defined as 2 weeks to 5 months post-onset
b) Stroke involving the anterior circulation, confirmed by computed tomography or magnetic resonance imaging
c) Age between 40 and 65 years
d) Lower limb motor recovery corresponding to Brunnstrom stages IV–V
e) Functional Ambulation Category (FAC) score ≥ 3
f) Ability to walk independently, with or without assistive devices
g) Montreal Cognitive Assessment Indonesian version (MoCA-INA) score ≥ 20
h) Medically stable with controlled comorbidities

Exclusion Criteria

a) Severe expressive or receptive aphasia limiting task comprehension
b) Unstable cardiovascular or medical conditions contraindicating moderate-intensity exercise
c) Moderate to severe depression (Patient Health Questionnaire-9 ≥ 15)

Baseline characteristics

Baseline demographic and clinical characteristics will be recorded before randomization. These will include age, sex, time since stroke onset, stroke characteristics based on available neuroimaging and clinical records, lower-limb motor recovery according to the Brunnstrom stage, FAC, cognitive status assessed using MoCA-INA, use of walking aids, and relevant medical comorbidities. Baseline characteristics will be summarized separately for the DTOCT, TOCT, and DTT groups.

### 2.4. Sample size

This study aims to recruit 36 participants, with 12 allocated to each study arm. As a pilot and feasibility study, no formal power calculation based on a clinical efficacy outcome was performed. The sample size was informed by the rule-of-thumb proposed by Julious, which recommends 12 participants per group for pilot studies when limited prior information is available, considering feasibility and the precision of estimates of the mean and variance [18]. Sim and Lewis further emphasized that pilot study sample sizes should be justified according to the precision and efficiency required to inform the design of a subsequent definitive trial [19]. Consistent with guidance for randomized pilot and feasibility trials, the present sample is intended primarily to evaluate feasibility parameters, including recruitment, adherence, retention, and safety, and to provide preliminary estimates of outcome variability and intervention effects for planning a future adequately powered trial [20].

### 2.5. Recruitment

Participants will be recruited from patients receiving rehabilitation services at the study site. Screening will be conducted by the clinical team based on medical records and eligibility criteria.

Eligible participants will be approached by the research team and provided with detailed information regarding the study objectives, procedures, potential risks, and benefits. Written informed consent will be obtained prior to enrolment. Participants will be randomized using a computer-generated random allocation sequence in a 1:1:1 ratio with block randomization. The allocation sequence will be prepared by an independent investigator not involved in recruitment or outcome assessment. Allocation concealment will be maintained using sequentially numbered, opaque, sealed envelopes. The envelopes will be opened only after completion of baseline assessment and informed consent. Recruitment is anticipated to begin on August 1st, 2026, with study completion expected by October 30th, 2026. The study will use single masking at the participant level. Participants will not be informed of their specific intervention allocation during the study period. Because therapists are responsible for delivering the intervention protocols, therapist blinding is not feasible. Data analysis will be performed using de-identified datasets. To maximize participant retention, appointment reminders will be provided before each scheduled intervention session. Participants who discontinue the intervention will be encouraged to complete the post-intervention assessments whenever feasible. Reasons for withdrawal, missed sessions, and protocol deviations will be documented throughout the study and summarized descriptively.

### 2.6. Ethical considerations

Ethical approval for this study was obtained from the Research Ethics Committee of Rumkit Tk. II Dustira, Cimahi, Indonesia (Approval No. Etik.RSD/012/II/2026; February 23, 2026), with additional approval from the Health Research Ethics Committee of RSUD Welas Asih, West Java Province (Approval No. 3366/070/KEPK-RSUD.WelasAsih/V/2026; May 18, 2026). The study will be conducted in accordance with the principles of the International Conference on Harmonisation–Good Clinical Practice (ICH-GCP) and the ethical principles of the Declaration of Helsinki. All eligible participants will receive detailed information regarding the study objectives, procedures, potential risks and benefits, and their right to withdraw from the study at any time without affecting their medical care. Written informed consent will be obtained from all participants before enrolment by the investigators or trained research personnel. The informed consent form will include specific consent for blood collection and analysis of serum BDNF for the purposes of this study only. Any future use of biological specimens for ancillary research will require additional ethical approval and participant consent. Participant confidentiality will be strictly maintained, and all collected data will be anonymized and used exclusively for research purposes. The study will be conducted in accordance with the approved protocol and reported following the Standard Protocol Items: Recommendations for Interventional Trials (SPIRIT) guidelines (See S1 File) [21]. Standard operating procedures will be implemented for participant recruitment, intervention delivery, outcome assessment, adverse event reporting, data management, and statistical analysis. Regular monitoring will be undertaken by the research team to ensure protocol adherence and data quality throughout the study.

Owing to the pilot nature of the study, the short intervention period, and the minimal risks associated with the rehabilitation interventions, an independent Data Monitoring Committee has not been established. Participant safety will instead be monitored continuously by the investigators and treating rehabilitation physicians. Any serious adverse event related to the study intervention will be documented and reported to the institutional ethics committees in accordance with institutional regulations.

### 2.7. Protocol amendments

Any important modifications to the protocol that may affect participant safety, study procedures, eligibility criteria, intervention, outcome measures, or statistical analyses will require approval from the institutional Research Ethics Committees before implementation. Significant amendments will also be reflected in subsequent protocol publications where appropriate.

### 2.8. Interventions

All participants will undergo 12 supervised rehabilitation sessions over 4 weeks (three 60-minute sessions per week; see S2 File for detailed protocols), including warm-up and cool-down periods. Treatment duration and therapist contact will therefore be standardized across groups, although intervention content and active workload will differ according to the assigned protocol. Training intensity and task complexity will be progressively adjusted according to participant performance and tolerance, with physiological responses and perceived exertion monitored throughout the intervention. All interventions will be delivered by licensed rehabilitation therapists with experience in stroke rehabilitation and prior training in the study protocol.

#### 2.8.1. Experimental Group: DTOCT.

Participants in the DTOCT group will receive an integrated intervention combining task-oriented circuit training with concurrent cognitive challenges. Each session will consist of approximately 25–50 minutes of active circuit-based training, structured across five functional stations designed to simulate real-world activities.

Training Components:

a) **Dual-Task Walking**. Participants will perform straight-line walking (3–8 meters) while executing sequential cognitive tasks such as forward and backward number counting.
b) **Obstacle Walking**. Participants will step over obstacles (5–14 cm in height) while performing semantic memory tasks, such as naming object categories.
c) **Stair Navigation**. Participants will ascend and descend stairs (10–17 cm step height) while identifying colors and shapes.
d) **Walking with Navigation Tasks**. Participants will navigate an oval walking path (3–8 meters) while performing cognitive interference tasks, such as an incongruent Stroop test.
e) **Activities of Daily Living (ADL) Simulation**. Participants will walk while carrying objects (e.g., a cup of liquid or a shopping bag) to engage executive function and working memory.
f) **Training Progression**. Both physical intensity and cognitive load will be progressively increased on a weekly basis to maintain an optimal level of challenge and to promote adaptive neuroplastic responses.

#### 2.8.2. Active Comparator: TOCT.

Participants in the TOCT group will perform high-intensity, task-oriented circuit training without additional cognitive tasks. The protocol focuses exclusively on motor relearning and functional strengthening through repetitive, goal-directed activities. The TOCT program consists of six circuit stations:

a) Standing and reaching in multiple directions
b) Sit-to-stand transitions using chairs of varying heights
c) Anterior and posterior stepping onto blocks
d) Lateral stepping for balance and stability
e) Forward step-up training
f) Heel raises to improve gait push-off mechanics
g) Each station will be performed for 2.5–5 minutes, with short rest intervals between stations.

#### 2.8.3. Active Comparator: DTT.

Participants in the DTT group will perform dual-task walking without circuit-based physical training. The intervention focuses on cognitive–motor integration in a simplified walking context. Training Components:

Each session will include approximately 30 minutes of dual-task activity, divided into two sets, within the 60-minute session. Activities include:

a) **Backward Counting**. Continuous subtraction from a randomly selected three-digit number
b) **Stroop Task**. Identifying font colors or semantic meanings of incongruent words
c) **Memory Tasks**. Sequential recall of word lists or everyday items
e) **Verbal Fluency Tasks**. Generating words based on categories or letter cues
f) **Training Progression**. The progression of both motor and cognitive tasks will be strictly monitored using a performance-based rule. Progression to a higher level of task difficulty will occur only when the participant achieves a mastery level with an error rate of ≤30%. If the error rate exceeds 30% or if the participant demonstrates signs of excessive fatigue, the task difficulty will remain at the current level or be regressed to ensure motor learning and safety

### 2.9. Intervention fidelity and safety monitoring

To ensure consistency across intervention groups, all sessions will be supervised by trained therapists. Standardized protocols will be followed for each group. Training intensity will be monitored using heart rate, blood pressure, Borg Rating of Perceived Exertion (RPE). Adverse events, including falls, injuries, or medical complications occurring during the intervention sessions, will be documented throughout the study period. Adverse events will be summarized by intervention group, including the type and severity of the event and its assessed relationship to the study intervention, where applicable. Exercise will be interrupted or terminated if SpO_2_ decreases below 88%, blood pressure exceeds 180/110 mmHg, or participants develop excessive perceived exertion, chest pain, severe dizziness, or syncope. Perceived exertion will be assessed using the RPE scale (6–20 or 0–10, depending on the version used), with higher scores indicating greater perceived exertion.

### 2.10. Outcome measures

Outcome measures in this study are categorized into primary outcomes, which focus on feasibility and safety, and secondary outcomes, which evaluate clinical and neurobiological effects of the intervention.

#### 2.10.1. Primary outcomes.

Primary outcomes are assessed throughout the 4-week intervention period to determine the feasibility, tolerability, and safety of the intervention protocols [20,22–24].

a) **Adherence rate**. Adherence will be assessed as the percentage of scheduled intervention sessions attended by each participant during the 4-week intervention period. Each participant is scheduled to complete 12 sessions; therefore, adherence will be calculated as the number of sessions attended divided by 12 and expressed as a percentage.
b) Retention rate. Retention will be defined as the proportion of enrolled participants who complete the full 4-week intervention protocol and the post-intervention assessment. Participants who discontinue the intervention or do not complete the final assessment will be recorded as withdrawals, together with the reasons for discontinuation where available. Adherence and retention will be summarized separately for each intervention group, and reasons for withdrawal, missed sessions, and protocol deviations will be documented and reported descriptively.
c) **Adverse events**. Safety will be evaluated by recording the incidence of adverse events throughout the 4-week intervention period. Adverse events will include falls and other medical events occurring during the training sessions. All events will be documented during the intervention period to characterize the safety and tolerability of each training protocol.
d) **Heart rate**. Exercise heart rate will be monitored during each of the 12 intervention sessions as an indicator of cardiovascular demand and exercise tolerability. Mean exercise heart rate will be recorded in beats per minute (bpm) using a digital heart-rate monitor.
e) **Blood pressure**. Hemodynamic stability will be assessed by measuring systolic and diastolic blood pressure before and after each intervention session. Blood pressure will be recorded in millimeters of mercury (mmHg) throughout the 4-week intervention period to identify potentially unsafe hemodynamic responses to exercise.
f) **Rating of Perceived Exertion (RPE)**. Subjective physical effort and exercise tolerability will be assessed using the RPE scale. The scale ranges from 6 to 20, or 0–10 depending on the version used, with higher scores indicating greater perceived exertion. RPE will be recorded at the end of each circuit station during all 12 intervention sessions over the 4-week study period.

#### 2.10.2. Secondary outcomes.

a) **Walking endurance**. Walking endurance will be assessed using the 6-Minute Walk Test (6MWT), with the total distance walked over 6 minutes recorded in meters. The 6MWT has demonstrated excellent test–retest reliability in individuals after stroke, with a pooled intraclass correlation coefficient of approximately 0.98, and has shown good concurrent and construct validity with gait speed and functional mobility measures. Greater walking distances indicate better walking endurance and functional capacity [25].
b) **Gait speed**. Gait speed will be assessed using the 10-Meter Walk Test (10MWT) and expressed in meters per second (m/s). The 10MWT is widely used in stroke rehabilitation and has demonstrated good-to-excellent test–retest reliability and construct validity in individuals after stroke. Higher walking speed indicates better functional mobility [26,27].
c) **Walking adaptability**. Walking adaptability will be evaluated using the Standardized Walking Obstacle Course (SWOC), which assesses obstacle negotiation under normal walking, tray-carrying, and reduced-visual-input conditions. Outcomes include completion time, step count, and the number of errors. In individuals with stroke, SWOC completion time and step count have demonstrated very satisfactory-to-excellent intra-rater, inter-rater, and test–retest reliability (ICC 0.851–0.993), together with evidence of concurrent validity with established measures of functional mobility. Shorter completion times, fewer steps, and fewer errors indicate better walking adaptability [28].
d) **Serum Brain-Derived Neurotrophic Factor (BDNF)**. BDNF is involved in neuronal survival, synaptic plasticity, and motor learning and has been investigated as a peripheral biomarker associated with neuroplastic responses following stroke and physical exercise [15,29,30]. However, serum BDNF is an indirect surrogate measure and does not directly quantify central nervous system neuroplasticity. Its circulating concentration may also be influenced by biological and pre-analytical factors; accordingly, BDNF findings will be interpreted cautiously and in conjunction with clinical outcomes. For measurement, serum BDNF concentration will be assessed at baseline and post-intervention using a sandwich enzyme-linked immunosorbent assay (ELISA), with results expressed in picograms per milliliter (pg/mL). To minimize potential variation related to fasting status and time of day, all blood samples will be collected between 08:00 and 10:00 AM following an overnight fast. Post-intervention blood samples will be collected 48 hours after the final exercise session to reduce the influence of transient acute exercise-related changes in circulating BDNF. Following collection, blood samples will be allowed to clot for approximately 30 minutes at room temperature and then centrifuged at 2,000 × g for 10 minutes. The separated serum will be aliquoted and stored at −80°C until ELISA analysis. Baseline and post-intervention samples will be collected and processed using the same standardized procedures. Repeated freeze–thaw cycles will be avoided by storing serum samples in separate aliquots until analysis.

### 2.11. Quality control and data monitoring

All investigators and research personnel will receive standardized training regarding participant recruitment, intervention delivery, outcome assessment, data collection, and Good Clinical Practice (GCP) principles before study commencement. Source documents and case report forms will be reviewed periodically to ensure protocol adherence, data completeness, and data accuracy. Only authorized study personnel will have access to identifiable participant information. Regular monitoring will be conducted throughout the study to ensure compliance with the study protocol and institutional ethical requirements. The schedule of participant enrolment, intervention delivery, and outcome assessments is summarized in Fig 1.

**Fig 1:**
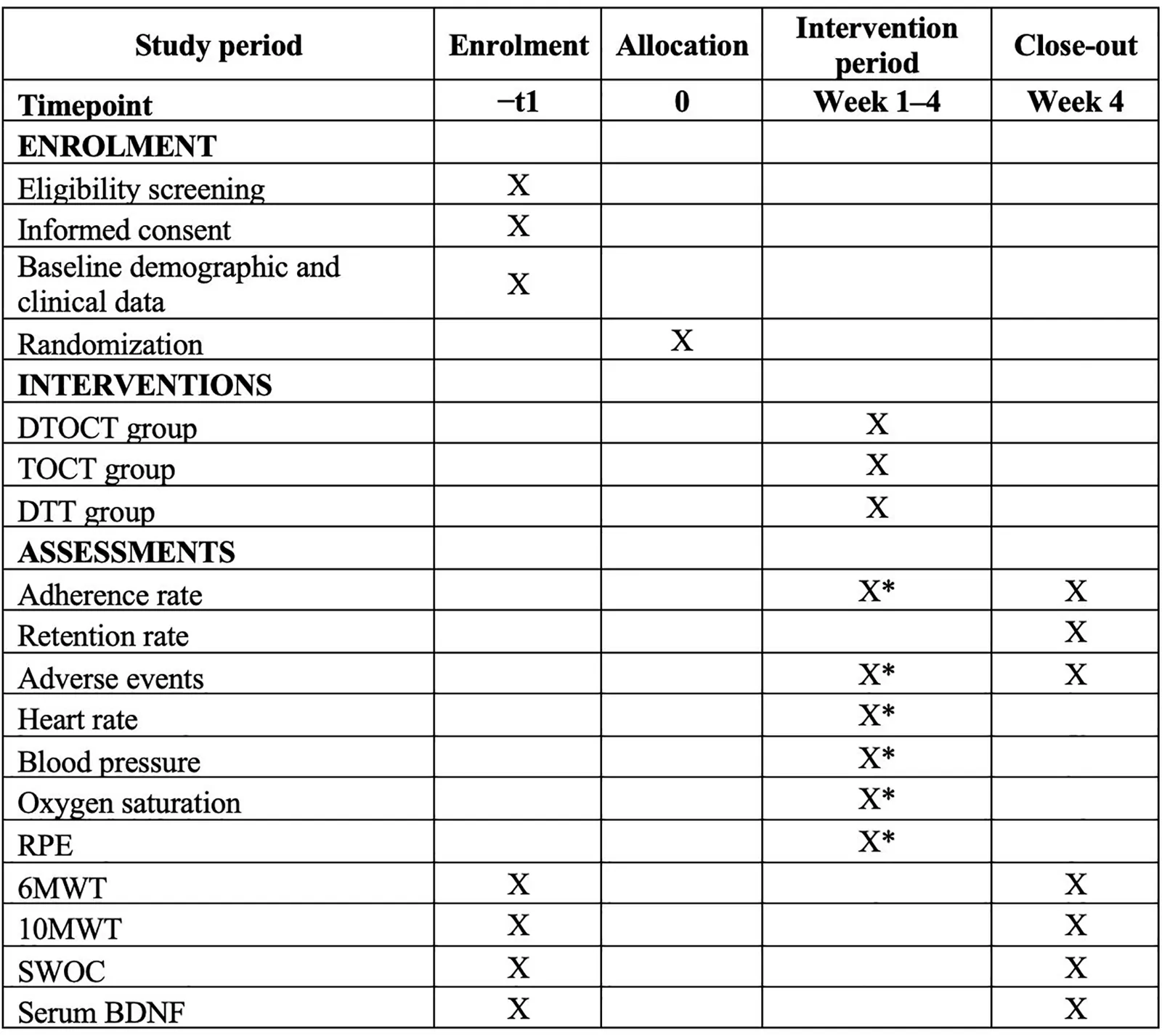
Schedule of participant enrolment, intervention delivery, and outcome assessments *Assessed during each intervention session (12 sessions over the 4-week intervention period).

### 2.12. Statistical analysis

All statistical analyses will be performed using IBM SPSS Statistics version 31 (IBM Corp., Armonk, NY, USA). As this is a pilot and feasibility trial, the primary objective of the analysis is to estimate feasibility, safety, and preliminary intervention effects rather than to establish definitive efficacy. Descriptive statistics will be used to summarize baseline demographic and clinical characteristics and outcome measures for each intervention group. Continuous variables will be presented as mean and standard deviation or median and interquartile range, depending on their distribution, whereas categorical variables will be presented as frequencies and percentages. Primary feasibility and safety outcomes, including adherence, retention, adverse events, heart rate, blood pressure, and RPE, will be summarized descriptively for each intervention group. Effect estimates and 95% confidence intervals will be reported where appropriate. Secondary clinical and neurobiological outcomes will be considered exploratory. Between-group differences at 4 weeks will primarily be analyzed using analysis of covariance (ANCOVA), with the post-intervention value as the dependent variable, intervention group as the fixed factor, and the corresponding baseline value as a covariate. Adjusted mean differences, effect sizes, and 95% confidence intervals will be reported. Model assumptions, including normality of residuals, homogeneity of variance, linearity, and influential observations, will be assessed. If these assumptions are substantially violated, appropriate transformation or a non-parametric rank-based or permutation-based ANCOVA approach will be considered.

Missing outcome data will not be imputed because of the exploratory pilot design. The extent and reasons for missing data, withdrawals, and protocol deviations will be reported descriptively. All statistical tests will be two-sided, with a p value <0.05 used for exploratory analyses. Interpretation will emphasize effect estimates and 95% confidence intervals rather than statistical significance. Where established and applicable, changes in walking outcomes will also be interpreted in relation to published thresholds for clinically meaningful change. For gait speed, an improvement of approximately 0.16 m/s has been proposed as a minimal clinically important difference (MCID) in individuals with subacute stroke [31]. For the 6MWT, published estimates vary according to baseline walking ability and the clinical anchor used; therefore, these thresholds will be used descriptively rather than as definitive efficacy criteria [32]. For SWOC outcomes, for which an established MCID is not available, published minimal detectable change values will be considered when interpreting whether observed changes exceed measurement error [28].

### 2.13. Data management

All data will be collected using standardized case report forms and recorded by trained research personnel. Participant data will be anonymized and assigned unique identification codes to ensure confidentiality. Data will be stored securely in password-protected electronic databases accessible only to authorized members of the research team. Hard copy documents, if any, will be stored in locked cabinets within the research facility. Quality control procedures will be implemented to ensure data accuracy, including double data entry verification and routine data audits. Any discrepancies identified during data checks will be resolved through review of source documents.

### 2.14. Safety monitoring

All adverse events will be evaluated by the clinical team and managed according to institutional clinical procedures (see S3 File). Participants may be withdrawn if continued participation is considered unsafe. Serious intervention-related adverse events will be reported to the relevant ethics committees in accordance with institutional requirements. No financial compensation is planned for trial-related harm. After study completion or withdrawal, participants will continue to receive standard rehabilitation care as determined by their treating physicians. No formal interim efficacy analysis or predefined efficacy stopping rule is planned because of the pilot nature and short duration of the study.

### 2.15. Trial status

At the time of this publication, the trial has been registered and is currently recruiting. The study is registered at ClinicalTrials.gov (ID: NCT07494357) and is being conducted according to protocol version 1.0 (IKFR-202603.01). Participant recruitment is anticipated to commence on August 1st, 2026, with enrollment of 36 participants planned. The primary completion date, including completion of the intervention and outcome assessments, is anticipated for October 30th, 2026. The overall study completion is also expected by October 30, 2026. Following completion of data collection, data analysis will be undertaken to evaluate the feasibility, safety, and preliminary efficacy of the DTOCT program in subacute stroke patients. The current study protocol and statistical analysis plan are available from the corresponding author upon reasonable request.

## 3. Discussion

This study protocol describes a pilot and feasibility trial designed to evaluate the implementation and preliminary effects of DTOCT in individuals with subacute stroke. The study addresses a critical gap in neurorehabilitation: many stroke survivors regain basic walking capacity in controlled clinical settings but continue to experience limitations in safe, efficient, and adaptive community ambulation. Community walking requires more than gait speed alone; it involves endurance, environmental negotiation, attentional control, and the ability to walk while performing concurrent tasks. Although the selected walking outcomes capture important components of mobility, improvements in structured clinical tests should not be interpreted as equivalent to improved community ambulation. Real-world walking involves greater environmental variability, divided attention, obstacle negotiation, navigation, and task adaptation that may not be fully represented by the 6MWT, 10MWT, or SWOC. Accordingly, these outcomes should be viewed as clinical indicators of walking capacity and adaptability rather than direct measures of community mobility. These limitations are particularly relevant in the presence of CMI, which can compromise motor or cognitive performance during concurrent tasks [33–35]. Whereas TOCT primarily develops functional motor capacity and DTT targets cognitive–motor integration [4,36–38], DTOCT combines these demands within a structured mobility framework. DTOCT integrates these components by embedding cognitive tasks into functional mobility exercises, thereby engaging motor, cognitive, and executive systems within a structured training framework. This approach is biologically plausible because dual-task walking after stroke is associated with increased activation of higher-order cortical regions, particularly the prefrontal cortex, while physical exercise may enhance neuroplasticity through systemic neurotrophic responses, including BDNF [29,30,39,40].

An important strength of this study is the combined use of clinical functional outcomes and a molecular biomarker (BDNF) to evaluate intervention effects. While previous studies have largely relied on functional assessments alone, the inclusion of BDNF provides complementary neurobiological information alongside functional outcomes. This dual-level evaluation may contribute to a more comprehensive understanding of how rehabilitation interventions influence both performance and neural adaptation. Accordingly, any change in serum BDNF should be interpreted as supportive neurobiological evidence rather than direct proof of enhanced central neuroplasticity. Its interpretation will therefore be considered alongside changes in walking performance and adaptability. The focus on the subacute phase further increases the clinical relevance of the study because this period is characterized by heightened responsiveness to rehabilitation. As much of the existing evidence has focused on chronic stroke, evaluating DTOCT earlier in recovery may help determine whether this approach warrants further investigation in a definitive trial.

As a pilot and feasibility trial, the primary objective of this study is not to establish definitive efficacy but to assess the practicality, safety, and acceptability of the intervention protocols. Several limitations should be acknowledged. First, the small sample size limits statistical precision and generalizability, while the short intervention period precludes evaluation of longer-term effects. Second, the absence of an untreated or usual-care control group limits the ability to isolate intervention-specific effects because spontaneous recovery and nonspecific rehabilitation effects may contribute to observed changes. Third, although treatment duration and therapist contact are standardized, differences in active workload, exercise intensity, and task specificity may also influence between-group effects. The single-center design further limits generalizability, and complete blinding is not feasible because therapists must know the assigned intervention. Distinct intervention characteristics may also influence participant expectations. In addition, recovery may be affected by intercurrent or iatrogenic complications, including healthcare-associated infections such as *Clostridioides difficile* infection, which can disrupt rehabilitation participation and functional recovery [41]. Taken together, these considerations warrant cautious interpretation of the exploratory findings.

In conclusion, this study will provide preliminary evidence regarding the feasibility and potential effectiveness of DTOCT as an integrated neurorehabilitation strategy for subacute stroke patients. The findings will provide feasibility metrics, outcome variability, and preliminary effect estimates to inform sample-size calculation, primary outcome selection, refinement of intervention dose and progression, and consideration of participant stratification in a subsequent adequately powered randomized controlled trial. By addressing both functional mobility and neuroplastic mechanisms, DTOCT represents an integrative rehabilitation approach whose feasibility, safety, and preliminary effects require further evaluation before any conclusions regarding comparative efficacy can be drawn.

## Supporting information

S1 File SPIRIT checklist. Completed SPIRIT checklist for the study protocol.
(DOCX)

S2a File Detailed intervention protocols (Bahasa). Detailed protocols for DTOCT, TOCT, and DTT, including session structure, task components, and progression procedures in Bahasa
(DOCX)

S2b File Detailed intervention protocols (English). Detailed protocols for DTOCT, TOCT, and DTT, including session structure, task components, and progression procedures in English.
(DOCX)

S3a File Safety monitoring protocol (Bahasa). Detailed procedures for physiological monitoring, exercise termination criteria, and adverse event management in Bahasa.
(DOCX)

S3b File Safety monitoring protocol (English). Detailed procedures for physiological monitoring, exercise termination criteria, and adverse event management in English.
(DOCX)
