# Supplementary material for: The effect of dual task-oriented circuit training on neuroplasticity and walking adaptability in subacute stroke patients: A pilot and feasibility study
Source: PLoS One. 2026 Oct 27;21(10):e0361255. doi: 10.1371/journal.pone.0361255 (PMC13712583; doi:10.1371/journal.pone.0361255)
Supplement: Supplementary file 2 [file pone.0361255.s002.docx]

**Protokol Standar Operasional Prosedur Rehabilitasi Neuroplastisitas: Implementasi Dual Task-Oriented Circuit Training pada Pasien Stroke Subakut**

## Transformasi Paradigma Rehabilitasi Stroke: Kerangka Kerja Neuroplastisitas dan Kemampuan Berjalan Adaptif

Stroke merupakan krisis kesehatan global yang menetap sebagai penyebab utama disabilitas jangka panjang pada orang dewasa di seluruh dunia, dengan estimasi 12 juta kejadian stroke baru setiap tahunnya.^1^ Di Indonesia, data Riset Kesehatan Dasar (RISKESDAS) menunjukkan prevalensi sebesar 10,9 per 1.000 penduduk, di mana fenomena ini semakin mengancam populasi usia produktif, dengan 66% kasus terjadi pada individu di bawah usia 70 tahun.^1^ Tantangan terbesar dalam neurorehabilitasi saat ini bukanlah sekadar mengembalikan kemampuan berjalan di lingkungan rumah sakit yang steril, melainkan menjembatani kesenjangan antara kapasitas fisik klinis dan kemampuan navigasi komunitas yang adaptif.^1^ Fenomena ini sering disebut sebagai *community ambulation gap*, di mana hanya sekitar 27% hingga 30% penyintas stroke yang mampu berintegrasi kembali sepenuhnya ke lingkungan luar yang dinamis meskipun telah menyelesaikan program rehabilitasi standar.^1^

Dasar neurologis dari hambatan ini terletak pada gangguan otomatisitas motorik. Berjalan, yang pada individu sehat dikendalikan secara otomatis oleh sirkuit subkortikal dan *central pattern generators*, pada pasien stroke berubah menjadi aktivitas yang sangat menuntut keterlibatan kortikal dan perhatian sadar.^1^ Kondisi ini memicu *Cognitive-Motor Interference* (CMI), sebuah fenomena di mana keterbatasan sumber daya perhatian pada otak yang mengalami lesi menyebabkan penurunan performa saat individu harus melakukan tugas motorik dan kognitif secara bersamaan, seperti berjalan sambil berbicara atau menghindari rintangan.^1^ Protokol *Dual Task-Oriented Circuit Training* (DTOCT) dikembangkan sebagai intervensi sinergis yang memanfaatkan "jendela emas" neuroplastisitas pada fase subakut (2 minggu hingga 5 bulan pasca-stroke) untuk mereorganisasi sirkuit neural melalui latihan berbasis tugas yang intensif dan tantangan kognitif yang terintegrasi.^1^

Implementasi protokol ini memerlukan ketelitian tinggi dari asisten peneliti dalam menjalankan setiap tahapan, mulai dari seleksi subjek hingga evaluasi biomarker molekuler seperti *Brain-Derived Neurotrophic Factor* (BDNF). BDNF merupakan neurotrofin krusial yang memfasilitasi kelangsungan hidup neuron, sinaptogenesis, dan *Long-Term Potentiation* (LTP), yang menjadi dasar biologis dari pembelajaran motorik dan memori.^1^ Melalui pemantauan yang ketat terhadap parameter klinis seperti *Six-Minute Walk Test* (6MWT) dan *Standardized Walking Obstacle Course* (SWOC), penelitian ini bertujuan untuk membuktikan bahwa integrasi beban kognitif dalam latihan sirkuit mampu memicu regulasi BDNF yang lebih superior dan meningkatkan adaptabilitas berjalan di dunia nyata.^1^

## Peran Strategis dan Kualifikasi Profesional Asisten Peneliti dalam Uji Klinis

Keberhasilan sebuah uji klinis, terutama yang melibatkan intervensi fisik intensitas tinggi pada populasi rentan seperti pasien stroke, sangat bergantung pada kompetensi dan etika kerja asisten peneliti. Seluruh prosedur dalam protokol ini harus dijalankan sesuai dengan prinsip *Good Clinical Practice* (GCP) atau Cara Uji Klinik yang Baik (CUKB) di Indonesia, yang menjamin bahwa data yang dihasilkan akurat, dapat dipercaya, serta hak dan kesejahteraan subjek terlindungi sepenuhnya.^7^ Asisten peneliti bertindak sebagai perpanjangan tangan peneliti utama dalam manajemen harian, pengumpulan data, dan monitoring keselamatan subjek.^7^

### Kompetensi Dasar dan Standar Etika

Asisten peneliti wajib memiliki pemahaman mendalam mengenai protokol penelitian, prinsip-prinsip etika Deklarasi Helsinki, dan prosedur operasional standar yang telah ditetapkan.^7^ Kualifikasi minimal yang diharapkan adalah gelar sarjana di bidang kesehatan (Kedokteran, Fisioterapi, atau Keperawatan) dengan sertifikasi CUKB yang masih berlaku.^8^ Kemampuan untuk melakukan anamnesis, pemeriksaan neurologis dasar, serta komunikasi terapeutik yang efektif sangat krusial dalam membangun kepercayaan dengan subjek dan keluarga.^14^

| **Area Tanggung Jawab** | **Deskripsi Tugas Asisten Peneliti** |
| --- | --- |
| **Administrasi & Regulasi** | Mengelola dokumen *Informed Consent*, menjaga kerahasiaan identitas subjek, dan memastikan ketersediaan logistik penelitian.^7^ |
| **Skrining & Rekrutmen** | Melakukan penyisiran rekam medis, verifikasi kriteria inklusi/eksklusi, dan memandu proses pendaftaran subjek.^13^ |
| **Pelaksanaan Intervensi** | Mengawasi sesi latihan, mencatat parameter fisiologis, dan menerapkan prinsip progresi 30% *error rule* secara tepat.^1^ |
| **Manajemen Data** | Melakukan input data ke *Case Report Form* (CRF), verifikasi akurasi data (*data cleaning*), dan pengarsipan sesuai prinsip ALCOA+.^22^ |

Setiap asisten peneliti harus menyadari bahwa mereka bekerja dengan populasi rentan yang mengalami disabilitas fisik dan potensi gangguan kognitif ringan hingga sedang. Oleh karena itu, empati dan kesabaran harus diutamakan tanpa mengorbankan ketegasan protokol.^1^ Segala bentuk kejadian tidak diinginkan (KTD) atau *Adverse Events* (AE) harus segera didokumentasikan dan dilaporkan kepada Peneliti Utama dalam waktu kurang dari 24 jam.^11^

## 1. Prosedur Operasional Standar: Skrining dan Seleksi Subjek

Tahapan skrining merupakan gerbang utama untuk memastikan keamanan subjek dan validitas internal penelitian. Asisten peneliti harus mengikuti langkah-langkah sistematis untuk memverifikasi kelayakan pasien stroke subakut sebelum dimasukkan ke dalam kelompok intervensi.^1^

### Kriteria Kelayakan Subjek

Pemilihan subjek didasarkan pada karakteristik klinis yang memungkinkan mereka untuk berpartisipasi dalam latihan sirkuit yang menantang secara motorik dan kognitif. Kriteria inklusi dan eksklusi didefinisikan secara ketat dalam tabel berikut untuk meminimalisir bias variabel pengganggu.^1^

| **Kategori** | **Kriteria Inklusi** | **Kriteria Eksklusi** |
| --- | --- | --- |
| **Diagnosis** | Stroke pertama, sirkulasi anterior (CT/MRI), fase subakut (2 mgg - 5 bln). | Stroke berulang, lesi serebelum, atau fase kronis (> 6 bulan). |
| **Demografi** | Usia 40–65 tahun, pria atau wanita. | Usia < 40 atau > 65 tahun. |
| **Motorik** | Brunnstrom Stage 4–5 (EB), FAC ≥ 3. | Brunnstrom < 4, tidak mampu berdiri tanpa bantuan maksimal. |
| **Kognitif** | MoCA-INA ≥ 18, mampu mengikuti instruksi. | Afasia berat, demensia vaskular lanjut, MoCA-INA < 18. |
| **Psikologis** | Kondisi stabil, bersedia menandatangani *Informed Consent*. | Depresi sedang-berat (PHQ-9 ≥ 15), gangguan psikotik. |
| **Kesehatan Umum** | Komorbiditas (HT, DM) terkontrol dan stabil. | Gagal jantung, angina tak stabil, aritmia berat, infeksi akut. |

### Alur Rekrutmen dan Persetujuan Setelah Penjelasan (PSP)

Proses rekrutmen dilakukan di unit rawat jalan rehabilitasi medik RS DUSTIRA. Asisten peneliti harus memberikan penjelasan yang komprehensif mengenai prosedur penelitian, termasuk jadwal latihan 3 kali seminggu selama 4 minggu, risiko kelelahan otot, serta manfaat potensial dalam peningkatan kemampuan berjalan.^1^ Subjek diberikan waktu yang cukup untuk berdiskusi dengan keluarga sebelum menandatangani formulir *Informed Consent*.

Setelah subjek setuju, asisten peneliti melakukan pengisian *Examination Form* (Lampiran 2) yang mencakup data dasar, riwayat penyakit, dan pemeriksaan fisik awal. Subjek kemudian dialokasikan ke salah satu kelompok (DTOCT, DTT, atau TOCT) menggunakan teknik *simple randomization* untuk menjamin keadilan alokasi dan menghindari bias pemilihan.

## 2. Protokol Pemeriksaan Pre-Intervensi (Baseline Assessment)

Pemeriksaan dasar dilakukan untuk memetakan kapasitas awal subjek dalam domain kognitif, motorik, dan biomarker molekuler. Penilaian ini harus dilakukan oleh evaluator yang terlatih dan, jika memungkinkan, disamarkan (*blinded*) dari alokasi kelompok intervensi subjek.

### Penilaian Kognitif dan Kesehatan Mental

Sebelum melakukan tes fisik, asisten peneliti wajib memastikan kondisi kognitif dan mental subjek memadai.

1. **MoCA-INA (Montreal Cognitive Assessment - Indonesia):** Digunakan untuk mendeteksi gangguan kognitif ringan. Asisten peneliti mengikuti panduan instruksi standar untuk menilai atensi, fungsi eksekutif, memori, dan orientasi
2. **PHQ-9 (Patient Health Questionnaire-9):** Untuk menskrining gejala depresi. Subjek dengan skor ≥ 15 (depresi sedang-berat) tidak dapat dilibatkan karena dapat mempengaruhi motivasi dan partisipasi dalam latihan intensif.

### Evaluasi Kapasitas Berjalan dan Mobilitas

Terdapat tiga instrumen utama yang digunakan untuk mengukur performa berjalan yang mencakup aspek ketahanan, kecepatan, dan adaptabilitas.

| **Instrumen** | **Prosedur Pelaksanaan** | **Parameter yang Dicatat** |
| --- | --- | --- |
| **6-Minute Walk Test (6MWT)** | Subjek berjalan secepat mungkin selama 6 menit di lintasan 30 meter. Istirahat diperbolehkan tetapi waktu tetap berjalan. | Jarak tempuh (meter), Borg Scale (sesak & lelah), Denyut Nadi, . |
| **10-Meter Walk Test (10MWT)** | Subjek berjalan 10 meter. Waktu diukur pada 6 meter tengah (zona 2–8m) untuk kecepatan nyaman dan cepat. | Waktu tempuh (detik), Kecepatan (), Penggunaan alat bantu jalan. |
| **Standardized Walking Obstacle Course (SWOC)** | Subjek menavigasi lintasan melengkung dengan rintangan (karpet, cone, rintangan 15cm) dalam 3 kondisi (normal, nampan, kacamata gelap). | Waktu penyelesaian, jumlah langkah, frekuensi tersandung atau keluar jalur. |

### Pemeriksaan Brunnstrom Stage Ekstremitas Bawah

Penilaian ini sangat teknis dan memerlukan asisten peneliti untuk melakukan evaluasi gerakan volunter secara spesifik untuk menentukan stadium pemulihan motorik.

**Stage 4–5:** Fokus pada kemampuan subjek melakukan gerakan yang menyimpang dari pola sinergi, seperti fleksi lutut melampaui 90 derajat dalam posisi duduk tanpa keterlibatan panggul berlebih. Buatkan video dengan instruksikan ke pasien untuk menggerakan secara aktif hip fleksi, knee fleksi-ekstensi dan ankle dorsifleksi pada kedua sisi ekstremitas bawah (bukan hanya sisi paretic). Serta buat video singkat berjalan dari sisi damping dan belakang untuk menilai Functional Ambulation category (FAC) dan Brunnstrom stage LE yang akan dilakukan oleh peneliti utama.

**Catatan:** pada kedua video, ekstremitas bawah pasien wajib terlihat dari atas lutut dan tanpa menggunakan alas kaki.

## Prosedur Laboratorium: Pengambilan dan Pengolahan Sampel BDNF

Pengukuran kadar *Brain-Derived Neurotrophic Factor* (BDNF) dalam serum berfungsi sebagai indikator biokimia dari neuroplastisitas yang diinduksi oleh latihan.

Asisten peneliti bertanggung jawab untuk memastikan pengambilan sampel dilakukan pada pukul 08.00-10.00 setelah puasa 8-12 jam sebelumnya. Untuk pemeriksaan baseline dilakukan pada saat belum dilakukan pemeriksaan apapun. Baru setelahnya dilakukan pemeriksaan baseline lain (10mwt,6mwt dan SWOC)

### Tahapan Flebotomi dan Pemrosesan Serum

Pengambilan sampel darah dilakukan dua kali, yaitu saat baseline (pre-test) dan setelah penyelesaian seluruh sesi intervensi (post-test).

. **Pengambilan Sampel:** Darah vena diambil sebanyak 5 mL menggunakan tabung tanpa antikoagulan (tutup merah) oleh tenaga phlebotomist yang tersertifikasi.
. **Pembekuan:** Darah didiamkan pada suhu ruang selama tepat 30 menit untuk memungkinkan pembentukan bekuan yang sempurna dan pelepasan BDNF dari trombosit.
. **Sentrifugasi:** Sampel disentrifugasi pada kecepatan 3000 rpm selama 15 menit untuk memisahkan serum dari komponen seluler.
. **Alikuot dan Penyimpanan:** Serum dipindahkan ke dalam *cryovial* (alikuot) sebanyak 0,5–1 mL per tabung untuk menghindari proses *freeze-thaw* berulang.

Simpan segera dalam freezer  di fasilitas laboratorium yang terkontrol.

Analisis dilakukan menggunakan teknik *Quantitative Sandwich Enzyme Immunoassay* dengan kit ELISA yang spesifik untuk Human BDNF. Asisten peneliti harus berkoordinasi dengan laboratorium mitra (seperti Prodia) untuk memastikan logistik pengiriman sampel dalam *dry ice* tetap terjaga suhunya di bawah  selama transit.

## 3. Implementasi Protokol Intervensi: Dual Task-Oriented Circuit Training (DTOCT)

Inti dari penelitian ini adalah pemberian intervensi DTOCT yang menggabungkan intensitas latihan fisik sirkuit dengan tantangan kognitif yang progresif. Protokol ini dirancang untuk memaksimalkan beban kerja sistem saraf pusat melalui tuntutan pembagian perhatian (*divided attention*).^1^

### Struktur Sesi Latihan

Setiap sesi latihan berlangsung selama 60 menit dengan frekuensi 3 kali per minggu selama 4 minggu.^1^ Asisten peneliti harus memastikan subjek menggunakan pakaian dan sepatu yang nyaman serta membawa alat bantu jalan jika diperlukan.

. **Pemanasan (5 Menit):** Melibatkan peregangan statis dan dinamis pada ekstremitas atas dan bawah untuk menyiapkan sistem muskuloskeletal.
. **Latihan Sirkuit (25–50 Menit):** Terdiri dari 5 stasiun yang masing-masing dijalankan selama 5–10 menit.
. **Pendinginan (5 Menit):** Peregangan ringan dan latihan pernapasan untuk mengembalikan frekuensi denyut nadi ke kondisi basal.

### Detail Stasiun Latihan DTOCT

| **Pos** | **Deskripsi Tugas Motorik** | **Tugas Kognitif Pendamping** | **Strategi Progresi (Mingguan)** |
| --- | --- | --- | --- |
| **1: Dual Task Walk** | Berjalan lurus 3–8 meter, dimulai dengan kaki paretik. | Mengucapkan urutan angka maju/mundur. | Tingkatkan jarak dan jumlah digit angka (misal 3 ke 5 digit). |
| **2: Obstacle Walk** | Melangkahi cone (5cm) dan hurdle (14cm). | Menyebutkan kategori benda (buah/hewan). | Tingkatkan jumlah rintangan dan persempit jarak antar rintangan. |
| **3: Stairs** | Naik-turun tangga (tinggi 10–17 cm). | Identifikasi warna dan bentuk geometri di tangga. | Tingkatkan tinggi anak tangga dan kompleksitas memori visual. |
| **4: Navigation** | Berjalan pada lintasan oval/berputar (3–8 m). | Identifikasi kata-warna (Stroop Task). | Gunakan stimulus tidak kongruen dan tambah rintangan permukaan (foam). |
| **5: ADL Stimulation** | Berjalan sambil membawa gelas berisi cairan/tas. | Menghafal daftar belanja secara berurutan. | Tambah volume cairan (100–150ml) dan berat tas belanja. |

### Prinsip Progresi: "30% Error Rule" dan Monitoring Intensitas

Untuk memastikan latihan tetap berada pada zona tantangan yang optimal bagi neuroplastisitas, asisten peneliti harus menerapkan aturan progresi yang ketat.

. **Aturan Kesalahan 30%:** Tingkat kesulitan tugas (baik motorik maupun kognitif) hanya boleh ditingkatkan ke level berikutnya jika tingkat kesalahan subjek pada level saat ini berada di bawah 30%.^1^ Jika kesalahan > 30%, subjek tetap pada level tersebut untuk memperkuat pembelajaran motorik.
. **Monitoring Borg Scale:** Intensitas latihan dipantau menggunakan Skala Borg (6–20). Target pada minggu ke-1 adalah skor 12–13 (*somewhat hard*), yang secara bertahap ditingkatkan hingga mencapai skor 15–16 (*hard*) pada minggu ke-4.
. **Keselamatan:** Denyut nadi dan tekanan darah diukur sebelum dan sesudah latihan. Hentikan latihan jika subjek mengeluh nyeri dada, pusing hebat, atau denyut nadi melebihi batas aman yang dihitung berdasarkan usia.

## 4. Perbandingan Kelompok Kontrol: DTT dan TOCT

Sebagai studi pilot RCT, protokol ini membandingkan DTOCT dengan dua metode standar lainnya untuk mengidentifikasi efek sinergis yang diharapkan.

### Protokol Dual Task Training (DTT)

Berbeda dengan kelompok sirkuit (DTOCT), kelompok DTT berfokus pada manajemen interferensi kognitif-motorik (CMI) saat berjalan di lintasan lurus dengan beban kognitif yang semakin kompleks.

**Struktur Sesi:**

- **Total Durasi:** 30 Menit (2 set dari 5 tugas kognitif).
- **Setiap Tugas:** 2 menit pelaksanaan + 1 menit istirahat antar tugas.
- **Kecepatan Jalan:** Pasien diminta berjalan pada kecepatan yang nyaman (comfortable walking speed).^1^

A. **Tabel Progresi Tugas Kognitif Mingguan (12 Sesi)**

**Aturan Emas:** Progresi level dilakukan pada Sesi 4, 7, dan 10 **HANYA JIKA** *error rate* kognitif pasien pada minggu sebelumnya < 30%.^1^

| **Pos** | **Minggu 1 (Sesi**  **1-3)** | **Minggu 2 (Sesi**  **4-6)** | **Minggu 3 (Sesi**  **7-9)** | **Minggu 4 (Sesi**  **10-12)** |
| --- | --- | --- | --- | --- |
| **1. Backward Counting** | Hitung mundur dari angka 3 digit (misal: 100) dengan pengurangan 1. | Hitung mundur angka 3 digit yang berbeda dengan pengurangan 1. | Hitung mundur angka 3 digit dengan pengurangan 3. | Hitung mundur angka 3 digit dengan pengurangan 7 (Level Maksimal). |
| **2. Stroop Task** | Identifikasi warna tinta pada 15 kata warna kongruen (misal: kata "Merah" tinta  merah). | Identifikasi warna tinta pada 15 kata warna tidak kongruen (Level 1). | Identifikasi makna kata pada 15 kata warna tidak kongruen (Level 2). | Acak antara identifikasi warna tinta dan makna kata (Level 3). |
| **3. Shopping List Recall** | Mengingat & menyebutkan 3 item belanja secara berurutan. | Mengingat & menyebutkan 4 item belanja secara berurutan. | Mengingat & menyebutkan 4 item belanja kategori berbeda. | Mengingat & menyebutkan 5 item belanja secara berurutan. |
| **4. Reverse Word Recall** | Menyebutkan 2 kata dalam urutan terbalik (misal: "Buku-Meja" \to  "Meja-Buku"). | Menyebutkan 3 kata dalam urutan terbalik. | Menyebutkan 4 kata dalam urutan terbalik. | Menyebutkan frasa kalimat pendek (5 kata) secara terbalik. |
| **5. Word Formation / Chain** | Menyebutkan hewan/buah yang dimulai dari 1 huruf tertentu dalam 2 menit. | Membuat rantai kata berdasarkan huruf terakhir (misal:  "Ikan-Nani-Itik"). | Rantai kata dengan batasan kategori tertentu (misal: Nama Kota/Negara). | Rantai kata dengan minimal 3 suku kata per kata. |

B. **Petunjuk Pelaksanaan untuk Research Asistant (RA)**

### 1. Monitoring Kognitif & Motorik

Selama 2 menit pelaksanaan tugas, RA wajib membawa lembar observasi untuk mencatat:

- **Jumlah Error:** Berapa kali pasien berhenti berjalan saat berpikir, salah menyebutkan angka/kata, atau kehilangan urutan.
- **Keputusan Progresi:** Hitung persentase kesalahan. Jika pasien salah > 6 kali dalam 20 percobaan kognitif (> 30%), maka minggu depan level kognitif **TIDAK** dinaikkan.[1]

### 2. Instruksi Verbal Standar

Gunakan instruksi yang konsisten untuk menghindari bias:

- ***“****Berjalanlah dengan kecepatan yang menurut Anda paling nyaman, sambil melakukan tugas yang saya berikan sesegera mungkin setelah saya katakan 'Mulai'."*

### 3. Penanganan Kelelahan

- Ukur **Skala Borg** setiap selesai 1 set (5 tugas).
- Jika Borg > 15 (*Hard*), perpanjang waktu istirahat menjadi 3 menit sebelum memulai set ke-2.

### 4. Keamanan Pasien

- RA harus berjalan setidaknya **setengah langkah di belakang** pasien pada sisi yang lemah (*paretic side*) untuk berjaga-jaga tanpa memengaruhi kecepatan jalan pasien (*avoid pacing*).

C. **Contoh Implementasi Sesi Minggu ke-4 (Level Maksimal)**

- **Tugas 1:** RA menyebutkan angka "457", pasien harus menjawab "450, 443, 436..." sambil terus berjalan.
- **Tugas 3:** RA menyebutkan "Bayam, Sabun, Gula, Telur, Susu". Pasien harus mengulangi daftar ini setelah berjalan 5 meter.[1]
- **Tugas 5:** Pasien melakukan *word chain*: "Gaja**h**" \to "Harima**u**" \to "Unt**a**" tanpa boleh berhenti melangkah.

### Protokol Task-Oriented Circuit Training (TOCT)

Protokol ini dirancang untuk memastikan beban latihan terus meningkat (*progressive overload*) guna memicu neuroplastisitas, namun tetap aman dengan mengikuti aturan progresi yang ketat.

**Prinsip Utama:** Progresi dilakukan setiap minggu (Sesi 4, 7, dan 10) **HANYA JIKA** subjek mampu menyelesaikan repetisi dengan tingkat kesalahan atau kegagalan motorik (misal: kehilangan keseimbangan ringan, langkah tidak sempurna) kurang dari 30%.^1^

A. **Spesifikasi Peralatan**

Berdasarkan bukti klinis efektivitas latihan *stepping* pada pasien stroke, kita menetapkan standar ketinggian berikut:

1. **Blok Latihan (*Step Box*):** Tinggi **10 cm** (rendah), **12 cm** (standar), dan **15 cm** (tinggi).
2. **Tangga Rehabilitasi:** Tinggi anak tangga **10 cm**, **15 cm**, dan **17 cm** (maksimal standar klinis).

B. **Tabel Progresi 6 Stasiun TOCT (4 Minggu)**

| **Nama Latihan** | **Minggu 1 (Sesi**  **1-3)** | **Minggu 2 (Sesi**  **4-6)** | **Minggu 3 (Sesi**  **7-9)** | **Minggu 4 (Sesi**  **10-12)** |
| --- | --- | --- | --- | --- |
| 1. **Standing & Reaching** | Menjangkau target setinggi bahu (depan/sampin g) | Target diagonal (menyilang) & lebih jauh. | Menjangkau ke arah lantai (fleksi batang tubuh). | Berdiri di atas *foam mat* (permukaan tidak stabil). |
| 2. **Sit-to-Stand** | Kursi standar (45 cm),  tangan di dada | Kursi lebih rendah (40 cm) tangan di dada | Kursi standar (45 cm) dengan tambahan alas foam empuk (2 cm), tangan di dada | Kursi rendah (40 cm) dengan tambahan alas foam empuk (2 cm), tangan di dada |
| 3. **Ant./Post. Stepping** | Melangkah arah depan, naik turun ke blok tinggi 10 cm | Melangkah arah depan, naik turun ke blok tinggi 12 cm | Melangkah arah depan, naik turun ke blok tinggi 15 cm | Melangkah arah depan, naik turun ke blok tinggi 15 cm dengan beban di pergelangan kaki (0.5kg) |
| 4. **Lateral Stepping** | Melangkah arah samping, naik turun ke blok tinggi 10 cm | Melangkah arah samping, naik turun ke blok tinggi 12 cm | Melangkah arah samping, naik turun ke blok tinggi 15 cm | Melangkah arah samping, naik turun cepat menyesuaikan bunyi metronom ke blok tinggi 12 cm |
| 5. **Forward**   **Step-Up** | Naik turun tangga tinggi 10 cm (berpegangan handrail) | Naik turun tangga tinggi 15 cm (berpegangan handrail) | Naik turun tangga tinggi 17 cm (berpegangan handrail) | Naik turun tangga tinggi 17 cm (tanpa berpegangan handrail) |
| 6. **Heel Raise** | Berdiri dan berjinjit (posisi tumit diangkat) di lantai datar dengan berpegangan | Berdiri dan berjinjit (posisi tumit diangkat) di lantai datar tanpa berpegangan | Berdiri di atas blok tinggi 12 cm dengan tumit menggantung | Berdiri 1 kaki dan berjinjit pada kaki sisi paretik dengan bantuan minimal |

C. **Instruksi Khusus untuk Asisten Peneliti (RA)**

### 1. Penerapan Aturan 30% Error

- Jika dalam 10 kali percobaan melangkah ke blok 12 cm, pasien tersandung atau memerlukan bantuan fisik > 3 kali, maka pada minggu berikutnya pasien TETAP di level 12 cm (jangan naik ke 15 cm).[1]

### 2. Ketinggian Tangga (Pos 5)

- Mulailah dengan tinggi **10 cm** pada minggu pertama untuk membangun kepercayaan diri subjek. Progresi ke 17 cm hanya diberikan bagi subjek dengan Functional Ambulation Category (FAC) yang baik.[1]

### 3. Ketinggian Blok (Pos 3 & 4 )

- Gunakan blok kayu atau *aerobic step* yang stabil. Pastikan seluruh telapak kaki pasien menapak sempurna di atas blok untuk menghindari cedera pergelangan kaki.

### 4. Monitoring Keamanan

- RA wajib berada di sisi paretik sebagai *spotter.*
- Evaluasi Skala Borg setiap perpindahan stasiun. Jika Borg > 16 (Hard), berikan istirahat tambahan hingga denyut nadi kembali ke rentang aman.

## 5. Protokol Pemeriksaan Pasca-Intervensi (Post-Test)

Setelah menyelesaikan total 12 sesi dalam 4 minggu, asisten peneliti menjadwalkan evaluasi akhir dalam jendela waktu 24–48 jam setelah sesi terakhir. Hal ini penting untuk menangkap efek adaptasi kronis tanpa terpengaruh oleh kelelahan akut pasca-latihan terakhir.

1. **Pengulangan Penilaian Klinis:** Lakukan kembali tes 6MWT, 10MWT, SWOC, dan Brunnstrom Stage menggunakan prosedur dan alat yang sama persis dengan saat baseline.^1^
2. **Evaluasi Kognitif:** Ulangi penilaian MoCA-INA untuk melihat apakah ada efek transfer dari latihan dual-task terhadap fungsi kognitif global.^5^
3. **Pengambilan Darah Post-Test:** Lakukan pengambilan darah untuk biomarker BDNF. Pastikan waktu pengambilan (jam) adalah 48 jam dari sesi latihan terakhir dan dilakukan pada pukul 08.00-10.00 tiap hari pengambilan
4. **Rekonsiliasi Data:** Periksa kelengkapan seluruh data dalam CRF. Pastikan tidak ada kolom yang kosong tanpa penjelasan yang sah.^22^

## 6. Manajemen Data, Dokumentasi, dan Jaminan Kualitas

Integritas hasil penelitian sangat bergantung pada cara asisten peneliti mengelola data mentah hingga menjadi informasi yang siap dianalisis. Seluruh proses dokumentasi harus mengikuti standar internasional yang ketat.^22^

### Penerapan Prinsip ALCOA+

### Setiap asisten peneliti wajib menerapkan prinsip ALCOA+ dalam setiap pencatatan data pada *Case Report Form* (CRF) maupun buku catatan harian (*logbook*).^22^

| **Unsur ALCOA+** | **Implementasi dalam Protokol** |
| --- | --- |
| **Attributable** | Setiap entri data harus menyertakan inisial pemeriksa dan tanggal.^22^ |
| **Legible** | Tulisan tangan harus jelas dan dapat dibaca oleh orang lain atau auditor.^22^ |
| **Contemporaneous** | Data dicatat pada saat pemeriksaan berlangsung, bukan ditunda hingga akhir hari.^22^ |
| **Original** | Pencatatan dilakukan pada formulir asli. Jangan gunakan kertas coretan yang kemudian disalin.^22^ |
| **Accurate** | Nilai yang dicatat harus sesuai dengan hasil pengukuran asli tanpa manipulasi.^22^ |
| **Complete & Consistent** | Seluruh kolom formulir harus terisi. Gunakan unit pengukuran yang seragam.^22^ |

### Pengisian Logbook Harian Asisten Peneliti

*Logbook* merupakan bukti fisik aktivitas penelitian harian. Asisten peneliti harus mencatat:

- Waktu kedatangan dan kepulangan.^29^
- Nama subjek yang melakukan latihan dan nomor sesi.^30^
- Rincian kegiatan yang dilakukan (misalnya: "Melakukan 10MWT pada subjek 005").^30^
- Kendala teknis atau keluhan subjek selama sesi.^31^
- Tanda tangan pengawas atau peneliti utama secara berkala untuk validasi.^30^

### Keamanan dan Kerahasiaan Data

Sesuai dengan kode etik kedokteran, seluruh data subjek harus dianonimkan menggunakan kode unik (misalnya: S001, S002). Berkas fisik (CRF) disimpan dalam lemari terkunci, sedangkan data digital harus disimpan dalam komputer dengan perlindungan kata sandi yang hanya dapat diakses oleh tim peneliti inti.^7^ Penyimpanan data dilakukan minimal selama 5–10 tahun sesuai dengan regulasi nasional untuk kepentingan audit atau inspeksi CUKB di masa mendatang.^19^

Hasil dari penelitian ini diharapkan dapat memberikan kontribusi nyata bagi pengembangan ilmu rehabilitasi neuromuskuler berbasis neuroplastisitas di Indonesia. Dengan menunjukkan hubungan antara latihan motorik-kognitif terintegrasi dengan perubahan biomarker molekuler, protokol ini dapat menjadi standar baru dalam pemulihan kemampuan berjalan adaptif bagi pasien stroke subakut untuk kembali berfungsi secara mandiri di komunitas.^1^

Seluruh asisten peneliti diharapkan menjalankan protokol ini dengan penuh dedikasi, ketelitian, dan integritas ilmiah yang tinggi, demi tercapainya standar publikasi internasional bereputasi dan, yang terpenting, demi peningkatan kualitas hidup para penyintas stroke.^1^

#### References

1. RDDU non final.pdf
2. Differential Effects of Cognitive vs. Motor Dual-Task Training in Stroke Rehabilitation: A Precision-Focused Meta-Analysis | medRxiv, accessed March 29, 2026, [https://www.medrxiv.org/content/10.64898/2026.01.23.26344517v1.full-text](https://www.google.com/url?q=https://www.medrxiv.org/content/10.64898/2026.01.23.26344517v1.full-text&sa=D&source=editors&ust=1774754033760128&usg=AOvVaw2GSlVYCqDhxBj9Cwkq7hwQ)
3. Dual Task Effects on Speed and Accuracy During Cognitive and Upper Limb Motor Tasks in Adults With Stroke Hemiparesis - PMC, accessed March 29, 2026, [https://pmc.ncbi.nlm.nih.gov/articles/PMC8250862/](https://www.google.com/url?q=https://pmc.ncbi.nlm.nih.gov/articles/PMC8250862/&sa=D&source=editors&ust=1774754033761107&usg=AOvVaw2OJp_vBK6pZVhuOjpUk8qv)
4. Upper Extremity-Cognitive Dual-Task Capacity Post-Stroke - PMC - NIH, accessed March 29, 2026, [https://pmc.ncbi.nlm.nih.gov/articles/PMC12065953/](https://www.google.com/url?q=https://pmc.ncbi.nlm.nih.gov/articles/PMC12065953/&sa=D&source=editors&ust=1774754033761852&usg=AOvVaw2g82Qwn-JDTATmGBlS9F32)
5. A meta-analysis of the effects of dual-task training on cognitive function in stroke patients, accessed March 29, 2026, [https://www.frontiersin.org/journals/neurology/articles/10.3389/fneur.2025.1417364/full](https://www.google.com/url?q=https://www.frontiersin.org/journals/neurology/articles/10.3389/fneur.2025.1417364/full&sa=D&source=editors&ust=1774754033762848&usg=AOvVaw3zRinpT1rE7Poqh8rJBlNJ)
6. Comparing the effectiveness of dual-task and single-task training on walking function in stroke recovery: A systematic review and meta-analysis - PMC, accessed March 29, 2026, [https://pmc.ncbi.nlm.nih.gov/articles/PMC11902943/](https://www.google.com/url?q=https://pmc.ncbi.nlm.nih.gov/articles/PMC11902943/&sa=D&source=editors&ust=1774754033763848&usg=AOvVaw0BbpIVdpoRwhXCbcDWZ1RG)
7. Standard Operating Procedure (SOP) - National Institute on Drug Abuse, accessed March 29, 2026, [https://nida.nih.gov/sites/default/files/nida_gcp_training_sop.pdf](https://www.google.com/url?q=https://nida.nih.gov/sites/default/files/nida_gcp_training_sop.pdf&sa=D&source=editors&ust=1774754033764659&usg=AOvVaw0ZriELHSY76oKJ2dEsEv67)
8. Pedoman Cara Uji Klinik yang Baik (CUKB) di Indonesia, accessed March 29, 2026, [https://id.gmp-platform.com/app/uploads/2020/08/Pedoman-Cara-Uji-Klinik-Yang-Baik-Di-Indonesia.pdf](https://www.google.com/url?q=https://id.gmp-platform.com/app/uploads/2020/08/Pedoman-Cara-Uji-Klinik-Yang-Baik-Di-Indonesia.pdf&sa=D&source=editors&ust=1774754033765525&usg=AOvVaw2mP6RfRQ9mUJMoq9CSzN-y)
9. PERATURAN KEPALA BADAN PENGAWAS OBAT DAN MAKANAN REPUBLIK INDONESIA NOMOR 9 TAHUN 2014 TENTANG TATA LAKSANA PERSETUJUAN UJI KLIN - KEP UPNVJ, accessed March 29, 2026, [https://kep.upnvj.ac.id/assets/referensi/10.%20tata%20laksana%20persetujuan%20uji%20klinik%202014.pdf](https://www.google.com/url?q=https://kep.upnvj.ac.id/assets/referensi/10.%20tata%20laksana%20persetujuan%20uji%20klinik%202014.pdf&sa=D&source=editors&ust=1774754033766498&usg=AOvVaw3eo6PEUzhzHfD_wXke628y)
10. Good Clinical Practice Training | Grants & Funding, accessed March 29, 2026, [https://grants.nih.gov/policy-and-compliance/policy-topics/clinical-trials/good-clinical-training](https://www.google.com/url?q=https://grants.nih.gov/policy-and-compliance/policy-topics/clinical-trials/good-clinical-training&sa=D&source=editors&ust=1774754033767219&usg=AOvVaw3B8k77VdCU9xzaWDYQRk-m)
11. BADAN PENGAWAS OBAT DAN MAKANAN KEPUTUSAN KEPALA BADAN PENGAWAS OBAT DAN MAKANAN REPUBLIK INDONESIA NOMOR 02002/SK/KBPOM TENTANG - JDIH Badan POM, accessed March 29, 2026, [https://jdih.pom.go.id/download/rule/875/02002%7CSK%7CKBPOM/2001](https://www.google.com/url?q=https://jdih.pom.go.id/download/rule/875/02002%7CSK%7CKBPOM/2001&sa=D&source=editors&ust=1774754033767806&usg=AOvVaw0Qh1vyGG4NPSKNmFs0qFZf)
12. KODE ETIK KEDOKTERAN INDONESIA DAN PEDOMAN PELAKSANAAN KODE ETIK KEDOKTERAN INDONESIA, accessed March 29, 2026, [https://luk.staff.ugm.ac.id/atur/sehat/Kode-Etik-Kedokteran.pdf](https://www.google.com/url?q=https://luk.staff.ugm.ac.id/atur/sehat/Kode-Etik-Kedokteran.pdf&sa=D&source=editors&ust=1774754033768320&usg=AOvVaw3NaDHZmSoGy5PMukDP7Wa6)
13. Cara menjadi Asisten Peneliti - Keterampilan & Deskripsi Pekerjaan – Jobstreet, accessed March 29, 2026, [https://id.jobstreet.com/id/career-advice/role/research-assistant](https://www.google.com/url?q=https://id.jobstreet.com/id/career-advice/role/research-assistant&sa=D&source=editors&ust=1774754033768903&usg=AOvVaw2iiuS2zEt76Rw0hswCwbmJ)
14. pedoman akademik program studi dokter spesialis neurologi - FK UNPAD, accessed March 29, 2026, [https://fk.unpad.ac.id/wp-content/uploads/2024/05/Pedoman-Akademik-Neurologi.pdf](https://www.google.com/url?q=https://fk.unpad.ac.id/wp-content/uploads/2024/05/Pedoman-Akademik-Neurologi.pdf&sa=D&source=editors&ust=1774754033769486&usg=AOvVaw1zWbuE0k_DmSmjwzese-Jp)
15. Kode Etik Kedokteran Indonesia - Equals.id, accessed March 29, 2026, [https://equalsid.or.id/dokumen/101124182354_KODEKI-Tahun-2012.pdf%20%C2%B7%20versi%201.pdf](https://www.google.com/url?q=https://equalsid.or.id/dokumen/101124182354_KODEKI-Tahun-2012.pdf%20%C2%B7%20versi%201.pdf&sa=D&source=editors&ust=1774754033769988&usg=AOvVaw1bMIljPcArZH_l2pHgDL-b)
16. Clinical Research SOPs - Research Quality and Compliance - The University of Utah, accessed March 29, 2026, [https://qualitycompliance.research.utah.edu/sop-library/](https://www.google.com/url?q=https://qualitycompliance.research.utah.edu/sop-library/&sa=D&source=editors&ust=1774754033770460&usg=AOvVaw1kj8LFOMTH5thpjNDAXHvG)
17. SOP #104 - Conduct of Clinical Study - Kent State University, accessed March 29, 2026, [https://www-s3-live.kent.edu/s3fs-root/s3fs-public/file/SOP_104_ClinicalStudyConduct.docx?VersionId=jnO4I1JG8vZ4ZMmSTXK5RaYBGOc57sEV](https://www.google.com/url?q=https://www-s3-live.kent.edu/s3fs-root/s3fs-public/file/SOP_104_ClinicalStudyConduct.docx?VersionId=jnO4I1JG8vZ4ZMmSTXK5RaYBGOc57sEV&sa=D&source=editors&ust=1774754033771148&usg=AOvVaw0LYuhgOAuOWHKthyd-t-JQ)
18. SOPs for Clinical Research | Institute for Global Health and Infectious Diseases, accessed March 29, 2026, [https://globalhealth.unc.edu/research/ighid-research-management/sops-for-clinical-research/](https://www.google.com/url?q=https://globalhealth.unc.edu/research/ighid-research-management/sops-for-clinical-research/&sa=D&source=editors&ust=1774754033771788&usg=AOvVaw1Qi_fOCR3nP-89QOssBKUp)
19. Standard Operating Procedures (SOPs) in Clinical Trials | Credevo Articles, accessed March 29, 2026, [https://credevo.com/articles/2023/01/05/standard-operating-procedures-sops-in-clinical-trials/](https://www.google.com/url?q=https://credevo.com/articles/2023/01/05/standard-operating-procedures-sops-in-clinical-trials/&sa=D&source=editors&ust=1774754033772413&usg=AOvVaw3w9ePiM3Udc1FGSOQPX3da)
20. KEPUTUSAN MENTERI KESEHATAN REPUBLIK INDONESIA NOMOR HK.01.07/MENKES/1458/2023 TENTANG PENYELENGGARAAN PENELITIAN KLINIK DI RU, accessed March 29, 2026, [https://keslan.kemkes.go.id/unduhan/fileunduhan_1690693655_545916.pdf](https://www.google.com/url?q=https://keslan.kemkes.go.id/unduhan/fileunduhan_1690693655_545916.pdf&sa=D&source=editors&ust=1774754033773190&usg=AOvVaw0Do-EUPfKTmxP8W0lBJYIz)
21. PROSEDUR OPERASIONAL BAKU (SOP) - SIMKEPKES - Universitas Hasanuddin, accessed March 29, 2026, [https://simkepkes.med.unhas.ac.id/assets/uploads/media/02a56e16eef6473c6300aaf1cd8735b7.pdf](https://www.google.com/url?q=https://simkepkes.med.unhas.ac.id/assets/uploads/media/02a56e16eef6473c6300aaf1cd8735b7.pdf&sa=D&source=editors&ust=1774754033774064&usg=AOvVaw1oOJetiXM-m0hacdbhXCtH)
22. GCP Guidelines Mastery: Essential Practices for Clinical Trials - CCRPS, accessed March 29, 2026, [https://ccrps.org/clinical-research-blog/gcp-guidelines-mastery-essential-practices-for-clinical-trials](https://www.google.com/url?q=https://ccrps.org/clinical-research-blog/gcp-guidelines-mastery-essential-practices-for-clinical-trials&sa=D&source=editors&ust=1774754033774804&usg=AOvVaw22YvK8GrRohFTlcY8CThfS)
23. ct-310 clinical research data management - University of South Alabama, accessed March 29, 2026, [https://www.southalabama.edu/colleges/com/research/resources/ct310clinicaldatamanagement.pdf](https://www.google.com/url?q=https://www.southalabama.edu/colleges/com/research/resources/ct310clinicaldatamanagement.pdf&sa=D&source=editors&ust=1774754033775545&usg=AOvVaw1aAL7-ipEl51_WUp3eUA1V)
24. Effective Data Collection & Management for Research Assistants - CCRPS, accessed March 29, 2026, [https://ccrps.org/clinical-research-blog/effective-data-collection-amp-management-for-research-assistants](https://www.google.com/url?q=https://ccrps.org/clinical-research-blog/effective-data-collection-amp-management-for-research-assistants&sa=D&source=editors&ust=1774754033776276&usg=AOvVaw1VxYYGTlpkcwh-xZoirXqB)
25. Pedoman dan Standar Etik Penelitian dan Pengembangan Kesehatan Nasional - KEPKN, accessed March 29, 2026, [https://kepkn.kemkes.go.id/assets/img/keppkn/file/BUKU_PEDOMAN_DAN_STANDAR_ETIK_PENELITIAN_DAN_PENGEMBANGAN_KESEHATAN_NASIONAL(KEPKN).pdf](https://www.google.com/url?q=https://kepkn.kemkes.go.id/assets/img/keppkn/file/BUKU_PEDOMAN_DAN_STANDAR_ETIK_PENELITIAN_DAN_PENGEMBANGAN_KESEHATAN_NASIONAL(KEPKN).pdf&sa=D&source=editors&ust=1774754033777150&usg=AOvVaw3jSFSiYB4JJImWFfWFW2YX)
26. Standard Operating Procedures for Clinical Research at USF Health CRC QUALITY CONTROL RESPONSIBILITY, accessed March 29, 2026, [https://health.usf.edu/-/media/v3/usf-health/medicine/Research/OCR/Standard-Operating-Procedures/SOP_601_QualityControl.ashx](https://www.google.com/url?q=https://health.usf.edu/-/media/v3/usf-health/medicine/Research/OCR/Standard-Operating-Procedures/SOP_601_QualityControl.ashx&sa=D&source=editors&ust=1774754033778164&usg=AOvVaw3G6VErLXqPyvZUez18Jb5R)
27. PROSEDUR OPERASIONAL BAKU (POB) - E-Komisi Etik Penelitian Universitas Padjadjaran, accessed March 29, 2026, [https://admkep.unpad.ac.id/sop/POB_2022.pdf](https://www.google.com/url?q=https://admkep.unpad.ac.id/sop/POB_2022.pdf&sa=D&source=editors&ust=1774754033778838&usg=AOvVaw2A01vWhHCZgrIcfhIb5xSs)
28. SOPs BIRAC CTN Clinical Research Secretariat - National Cancer Grid, accessed March 29, 2026, [https://www.ncgindia.org/assets/ncg-research/sops/sops-birac-ctn-clinical-research-secretariat.pdf](https://www.google.com/url?q=https://www.ncgindia.org/assets/ncg-research/sops/sops-birac-ctn-clinical-research-secretariat.pdf&sa=D&source=editors&ust=1774754033779663&usg=AOvVaw3_VB1wyJuF-JjVX5sDs-JQ)
29. LOGBOOK - Bagian IKM-KP FK UNS, accessed March 29, 2026, [http://ikm.fk.uns.ac.id/wp-content/uploads/2015/10/Log-Book.doc](https://www.google.com/url?q=http://ikm.fk.uns.ac.id/wp-content/uploads/2015/10/Log-Book.doc&sa=D&source=editors&ust=1774754033780335&usg=AOvVaw0Fm47OGq6YhmG0GAhs3wAP)
30. Log Book Penelitian Kebidanan D-IV | PDF - Scribd, accessed March 29, 2026, [https://id.scribd.com/document/511081200/log-book](https://www.google.com/url?q=https://id.scribd.com/document/511081200/log-book&sa=D&source=editors&ust=1774754033780887&usg=AOvVaw2N4SqR241tegLoFTiHmGh9)
31. Contoh Logbook Penelitian Skripsi | PDF - Scribd, accessed March 29, 2026, [https://www.scribd.com/document/453815779/LOG-BOOK-PENELITIAN-docx](https://www.google.com/url?q=https://www.scribd.com/document/453815779/LOG-BOOK-PENELITIAN-docx&sa=D&source=editors&ust=1774754033781430&usg=AOvVaw1cVwXU-7RCR6gfQhFGoQPW)
32. 2.b Log Book Penelitian | PDF - Scribd, accessed March 29, 2026, [https://id.scribd.com/document/495364996/2-b-Log-Book-Penelitian](https://www.google.com/url?q=https://id.scribd.com/document/495364996/2-b-Log-Book-Penelitian&sa=D&source=editors&ust=1774754033781921&usg=AOvVaw3q-5Zk8I082SOKPw7u8Qxb)
33. Good Clinical Practice Research Guidelines Reviewed, Emphasis Given to Responsibilities of Investigators: Second Article in a Series - PMC, accessed March 29, 2026, [https://pmc.ncbi.nlm.nih.gov/articles/PMC2794015/](https://www.google.com/url?q=https://pmc.ncbi.nlm.nih.gov/articles/PMC2794015/&sa=D&source=editors&ust=1774754033782561&usg=AOvVaw2ynWfV0D-Cd1cHcCqsMKkz)
