# Supplementary material for: The effect of dual task-oriented circuit training on neuroplasticity and walking adaptability in subacute stroke patients: A pilot and feasibility study
Source: PLoS One. 2026 Oct 27;21(10):e0361255. doi: 10.1371/journal.pone.0361255 (PMC13712583; doi:10.1371/journal.pone.0361255)
Supplement: Supplementary file 3 [file pone.0361255.s003.docx]

**Standard Operating Procedure Protocol for Neuroplasticity Rehabilitation: Implementation of Dual Task-Oriented Circuit Training in Subacute Stroke Patients**

## Transforming the Stroke Rehabilitation Paradigm: A Neuroplasticity Framework for Adaptive Walking Ability

Stroke remains a persistent global health crisis and the leading cause of long-term disability among adults worldwide, with an estimated 12 million new stroke cases occurring each year.^1^ In Indonesia, data from the Basic Health Research (RISKESDAS) shows a prevalence of 10.9 per 1,000 population, with the condition increasingly threatening the working-age population, as 66% of cases occur in individuals under 70 years of age.^1^ The greatest challenge in neurorehabilitation today is not merely restoring walking ability within the sterile hospital environment, but bridging the gap between clinical physical capacity and adaptive community navigation ability.^1^ This phenomenon is often referred to as the *community ambulation gap*, in which only about 27% to 30% of stroke survivors are able to fully reintegrate into the dynamic outdoor environment despite having completed a standard rehabilitation program.^1^

The neurological basis of this limitation lies in impaired motor automaticity. Walking, which in healthy individuals is automatically controlled by subcortical circuits and *central pattern generators*, becomes, in stroke patients, an activity that heavily demands cortical involvement and conscious attention.^1^ This condition triggers *Cognitive-Motor Interference* (CMI), a phenomenon in which limited attentional resources in the lesioned brain cause performance to decline when an individual must perform motor and cognitive tasks simultaneously, such as walking while talking or avoiding obstacles.^1^ The *Dual Task-Oriented Circuit Training* (DTOCT) protocol was developed as a synergistic intervention that takes advantage of the neuroplasticity "golden window" during the subacute phase (2 weeks to 5 months post-stroke) to reorganize neural circuits through intensive task-based training combined with integrated cognitive challenges.^1^

Implementing this protocol requires a high level of precision from research assistants in carrying out every stage, from subject selection to the evaluation of molecular biomarkers such as *Brain-Derived Neurotrophic Factor* (BDNF). BDNF is a crucial neurotrophin that facilitates neuronal survival, synaptogenesis, and *Long-Term Potentiation* (LTP), which forms the biological basis of motor learning and memory.^1^ Through close monitoring of clinical parameters such as the *Six-Minute Walk Test* (6MWT) and the *Standardized Walking Obstacle Course* (SWOC), this study aims to demonstrate that integrating cognitive load into circuit training can trigger superior BDNF regulation and improve real-world walking adaptability.^1^

## Strategic Role and Professional Qualifications of Research Assistants in Clinical Trials

The success of a clinical trial, especially one involving high-intensity physical intervention in a vulnerable population such as stroke patients, depends heavily on the competence and work ethic of the research assistants. All procedures in this protocol must be carried out in accordance with the principles of *Good Clinical Practice* (GCP), known in Indonesia as Cara Uji Klinik yang Baik (CUKB), which ensures that the data generated is accurate and trustworthy, and that the rights and welfare of subjects are fully protected.^7^ Research assistants act as an extension of the principal investigator in day-to-day management, data collection, and subject safety monitoring.^7^

### Core Competencies and Ethical Standards

Research assistants must have an in-depth understanding of the research protocol, the ethical principles of the Declaration of Helsinki, and the established standard operating procedures.^7^ The minimum expected qualification is a bachelor’s degree in a health-related field (Medicine, Physiotherapy, or Nursing) with a valid CUKB (GCP) certification.^8^ The ability to take a patient history, perform basic neurological examinations, and communicate therapeutically is crucial for building trust with subjects and their families.^14^

| **Area of Responsibility** | **Description of Research Assistant Duties** |
| --- | --- |
| **Administration & Regulatory** | Manage *Informed Consent* documents, maintain the confidentiality of subject identities, and ensure the availability of research logistics.^7^ |
| **Screening & Recruitment** | Review medical records, verify inclusion/exclusion criteria, and guide the subject enrollment process.^13^ |
| **Intervention Delivery** | Supervise training sessions, record physiological parameters, and accurately apply the 30% *error rule* progression principle.^1^ |
| **Data Management** | Enter data into the *Case Report Form* (CRF), verify data accuracy (*data cleaning*), and archive it in accordance with the ALCOA+ principles.^22^ |

Every research assistant must recognize that they are working with a vulnerable population experiencing physical disability and potential mild-to-moderate cognitive impairment. Empathy and patience must therefore be prioritized without compromising strict adherence to the protocol.^1^ Any form of adverse event (AE), or *Adverse Events* (AE), must be immediately documented and reported to the Principal Investigator within less than 24 hours.^11^

## 1. Standard Operating Procedure: Subject Screening and Selection

The screening stage serves as the main gateway to ensuring subject safety and the internal validity of the study. Research assistants must follow systematic steps to verify the eligibility of subacute stroke patients before enrolling them in an intervention group.^1^

### Subject Eligibility Criteria

Subject selection is based on clinical characteristics that allow participation in a motor- and cognitively-challenging circuit training program. The inclusion and exclusion criteria are strictly defined in the table below to minimize confounding variable bias.^1^

| **Category** | **Inclusion Criteria** | **Exclusion Criteria** |
| --- | --- | --- |
| **Diagnosis** | First-ever stroke, anterior circulation (CT/MRI), subacute phase (2 wks - 5 mos). | Recurrent stroke, cerebellar lesion, or chronic phase (> 6 months). |
| **Demographics** | Age 40–65 years, male or female. | Age < 40 or > 65 years. |
| **Motor Function** | Brunnstrom Stage 4–5 (LE), FAC ≥ 3. | Brunnstrom < 4, unable to stand without maximal assistance. |
| **Cognitive** | MoCA-INA ≥ 18, able to follow instructions. | Severe aphasia, advanced vascular dementia, MoCA-INA < 18. |
| **Psychological** | Stable condition, willing to sign the *Informed Consent* form. | Moderate-to-severe depression (PHQ-9 ≥ 15), psychotic disorder. |
| **General Health** | Controlled and stable comorbidities (hypertension, diabetes). | Heart failure, unstable angina, severe arrhythmia, acute infection. |

### Recruitment Flow and Informed Consent Process

The recruitment process takes place at the outpatient medical rehabilitation unit of DUSTIRA Hospital. Research assistants must provide a comprehensive explanation of the study procedures, including the training schedule of 3 sessions per week for 4 weeks, the risk of muscle fatigue, and the potential benefits for improving walking ability.^1^ Subjects are given sufficient time to discuss with their families before signing the *Informed Consent* form.

Once the subject agrees, the research assistant completes the *Examination Form* (Appendix 2), which covers baseline data, medical history, and an initial physical examination. The subject is then allocated to one of the groups (DTOCT, DTT, or TOCT) using a *simple randomization* technique to ensure fair allocation and avoid selection bias.

## 2. Pre-Intervention Assessment Protocol (Baseline Assessment)

The baseline assessment is conducted to map the subject’s initial capacity in the cognitive, motor, and molecular biomarker domains. This assessment must be carried out by a trained evaluator and, where possible, blinded (*blinded*) to the subject’s intervention group allocation.

### Cognitive and Mental Health Assessment

Before conducting physical tests, research assistants must ensure that the subject’s cognitive and mental condition is adequate.

1. **MoCA-INA (Montreal Cognitive Assessment - Indonesia):** Used to detect mild cognitive impairment. Research assistants follow the standard instruction guide to assess attention, executive function, memory, and orientation.
2. **PHQ-9 (Patient Health Questionnaire-9):** Used to screen for depressive symptoms. Subjects with a score ≥ 15 (moderate-to-severe depression) cannot be included, as this may affect motivation and participation in intensive training.

### Evaluation of Walking Capacity and Mobility

Three main instruments are used to measure walking performance, covering the aspects of endurance, speed, and adaptability.

| **Instrument** | **Procedure** | **Recorded Parameters** |
| --- | --- | --- |
| **6-Minute Walk Test (6MWT)** | The subject walks as fast as possible for 6 minutes along a 30-meter track. Resting is permitted, but the timer keeps running. | Distance covered (meters), Borg Scale (breathlessness & fatigue), Pulse rate, . |
| **10-Meter Walk Test (10MWT)** | The subject walks 10 meters. Time is measured over the middle 6 meters (2–8 m zone) for both comfortable and fast walking speed. | Time (seconds), Speed (), Use of a walking aid. |
| **Standardized Walking Obstacle Course (SWOC)** | The subject navigates a curved course with obstacles (carpet, cones, a 15 cm obstacle) under 3 conditions (normal, carrying a tray, wearing dark glasses). | Completion time, number of steps, frequency of tripping or veering off course. |

### Lower Extremity Brunnstrom Stage Assessment

This assessment is highly technical and requires the research assistant to specifically evaluate voluntary movement to determine the stage of motor recovery.

**Stage 4–5:** Focus on the subject’s ability to perform movement out of synergy, such as knee flexion beyond 90 degrees in a sitting position without excessive hip involvement. Record a video instructing the patient to actively perform hip flexion, knee flexion-extension, and ankle dorsiflexion on both lower extremities (not only the paretic side). Also record a short video of walking from the side and from behind to allow the principal investigator to assess the Functional Ambulation Category (FAC) and lower-extremity Brunnstrom stage.

**Note:** in both videos, the patient’s lower extremities must be visible from above the knee and without footwear.

## Laboratory Procedure: BDNF Sample Collection and Processing

Measurement of *Brain-Derived Neurotrophic Factor* (BDNF) serum levels serves as a biochemical indicator of exercise-induced neuroplasticity.

Research assistants are responsible for ensuring that sample collection is carried out between 08:00-10:00 after 8-12 hours of prior fasting. For the baseline examination, blood sampling is performed before any other assessment is conducted. Only afterward are the other baseline assessments performed (10MWT, 6MWT, and SWOC).

### Phlebotomy and Serum Processing Steps

Blood samples are collected twice: at baseline (pre-test) and after completion of all intervention sessions (post-test).

. **Sample Collection:** 5 mL of venous blood is drawn using a tube without anticoagulant (red top) by a certified phlebotomist.
. **Clotting:** The blood is left at room temperature for exactly 30 minutes to allow complete clot formation and the release of BDNF from platelets.
. **Centrifugation:** The sample is centrifuged at 3000 rpm for 15 minutes to separate the serum from cellular components.
. **Aliquoting and Storage:** The serum is transferred into a *cryovial* (aliquoted) at 0.5–1 mL per tube to avoid repeated *freeze-thaw* cycles.

Store immediately in a freezer at  in a controlled laboratory facility.

Analysis is performed using the *Quantitative Sandwich Enzyme Immunoassay* technique with an ELISA kit specific for Human BDNF. Research assistants must coordinate with partner laboratories (such as Prodia) to ensure that sample shipment logistics in *dry ice* maintain a temperature below  during transit.

## 3. Implementation of the Intervention Protocol: Dual Task-Oriented Circuit Training (DTOCT)

The core of this study is the delivery of the DTOCT intervention, which combines the intensity of physical circuit training with progressive cognitive challenges. This protocol is designed to maximize the workload on the central nervous system through the demands of divided attention (*divided attention*).^1^

### Training Session Structure

Each training session lasts 60 minutes, at a frequency of 3 times per week for 4 weeks.^1^ Research assistants must ensure the subject wears comfortable clothing and shoes and brings a walking aid if needed.

. **Warm-Up (5 Minutes):** Involves static and dynamic stretching of the upper and lower extremities to prepare the musculoskeletal system.
. **Circuit Training (25–50 Minutes):** Consists of 5 stations, each carried out for 5–10 minutes.
. **Cool-Down (5 Minutes):** Light stretching and breathing exercises to return the pulse rate to its baseline level.

### DTOCT Training Station Details

| **Station** | **Motor Task Description** | **Concurrent Cognitive Task** | **Progression Strategy (Weekly)** |
| --- | --- | --- | --- |
| **1: Dual Task Walk** | Walk in a straight line for 3–8 meters, starting with the paretic leg. | Recite a sequence of numbers forward/backward. | Increase the distance and the number of digits (e.g., from 3 to 5 digits). |
| **2: Obstacle Walk** | Step over a cone (5cm) and a hurdle (14cm). | Name items within a category (fruits/animals). | Increase the number of obstacles and narrow the spacing between them. |
| **3: Stairs** | Go up and down stairs (10–17 cm step height). | Identify colors and geometric shapes on the stairs. | Increase the step height and the complexity of the visual memory task. |
| **4: Navigation** | Walk along an oval/turning course (3–8 m). | Identify word-color pairs (Stroop Task). | Use incongruent stimuli and add surface obstacles (foam). |
| **5: ADL Stimulation** | Walk while carrying a cup filled with liquid or a bag. | Memorize a shopping list in sequence. | Increase the liquid volume (100–150ml) and the weight of the shopping bag. |

### Progression Principle: the "30% Error Rule" and Intensity Monitoring

To ensure training remains within the optimal challenge zone for neuroplasticity, research assistants must apply strict progression rules.

. **30% Error Rule:** The difficulty level of a task (motor or cognitive) may only be advanced to the next level if the subject’s error rate at the current level is below 30%.^1^ If the error rate exceeds > 30%, the subject remains at that level to reinforce motor learning.
. **Borg Scale Monitoring:** Training intensity is monitored using the Borg Scale (6–20). The target for week 1 is a score of 12–13 (*somewhat hard*), which is gradually increased to a score of 15–16 (*hard*) by week 4.
. **Safety:** Pulse rate and blood pressure are measured before and after each session. Stop the training if the subject reports chest pain, severe dizziness, or if the pulse rate exceeds the age-based safe limit.

## 4. Comparison of Control Groups: DTT and TOCT

As a pilot RCT study, this protocol compares DTOCT with two other standard methods to identify the expected synergistic effect.

### Dual Task Training (DTT) Protocol

Unlike the circuit-training group (DTOCT), the DTT group focuses on managing cognitive-motor interference (CMI) while walking along a straight path with progressively more complex cognitive loads.

**Session Structure:**

- **Total Duration:** 30 Minutes (2 sets of 5 cognitive tasks).
- **Each Task:** 2 minutes of execution + 1 minute rest between tasks.
- **Walking Speed:** Patients are asked to walk at a comfortable walking speed.^1^

A. **Weekly Cognitive Task Progression Table (12 Sessions)**

**Golden Rule:** Level progression takes place at Sessions 4, 7, and 10, **ONLY IF** *the cognitive* error rate of the patient in the previous week is < 30%.^1^

| **Station** | **Week 1 (Sessions**  **1-3)** | **Week 2 (Sessions**  **4-6)** | **Week 3 (Sessions**  **7-9)** | **Week 4 (Sessions**  **10-12)** |
| --- | --- | --- | --- | --- |
| **1. Backward Counting** | Count backward from a 3 digit number (e.g., 100) with subtraction of 1. | Count backward from a 3 digit different number, with subtraction of 1. | Count backward from a 3 digit number with subtraction of 3. | Count backward from a 3 digit number with subtraction of 7 (Maximum Level). |
| **2. Stroop Task** | Identify the ink color of 15 congruent color words (e.g., the word "Red" in  red ink). | Identify the ink color of 15 incongruent color words (Level 1). | Identify the meaning of 15 incongruent color words (Level 2). | Randomly alternate between identifying ink color and word meaning (Level 3). |
| **3. Shopping List Recall** | Remember and recite 3 shopping items in order. | Remember and recite 4 shopping items in order. | Remember and recite 4 shopping items from different categories. | Remember and recite 5 shopping items in order. |
| **4. Reverse Word Recall** | Say 2 words in reverse order (e.g., "Buku-Meja" \to  "Meja-Buku"). | Say 3 words in reverse order. | Say 4 words in reverse order. | Say a short sentence phrase (5 words) in reverse. |
| **5. Word Formation / Chain** | Name animals/fruits starting with a specific letter within 2 minutes. | Build a word chain based on the last letter of the previous word (e.g.,  "Ikan-Nani-Itik"). | Word chain with a category restriction (e.g., City/Country names). | Word chain with a minimum of 3 syllables per word. |

B. **Implementation Guidelines for the Research Assistant (RA)**

### 1. Cognitive & Motor Monitoring

During the 2-minute task execution, the RA must carry an observation sheet to record:

- **Number of Errors:** How many times the patient stops walking to think, says the wrong number/word, or loses track of the sequence.
- **Progression Decision:** Calculate the error percentage. If the patient makes more than 6 errors in 20 cognitive trials (> 30%), then the cognitive level for the following week is **NOT** increased.[1]

### 2. Standard Verbal Instructions

Use consistent instructions to avoid bias:

- ***“****Walk at whatever pace feels most comfortable to you, while performing the task I give you as soon as possible after I say 'Start'."*

### 3. Managing Fatigue

- Measure the **Borg Scale** after completing each set (5 tasks).
- If the Borg score is > 15 (*Hard*), extend the rest period to 3 minutes before starting the 2nd set.

### 4. Patient Safety

- The RA must walk at least **half a step behind** the patient, on the weak side (*paretic side*), to be ready to assist without influencing the patient’s walking speed (*avoid pacing*).

C. **Example Implementation of a Week 4 Session (Maximum Level)**

- **Task 1:** The RA says the number "457", the patient must respond "450, 443, 436..." while continuing to walk.
- **Task 3:** The RA says "Spinach, Soap, Sugar, Eggs, Milk". The patient must repeat this list after walking 5 meters.[1]
- **Task 5:** The patient performs a *word chain*: "Gaja**h**" \to "Harima**u**" \to "Unt**a**" without stopping.

### Task-Oriented Circuit Training (TOCT) Protocol

This protocol is designed to ensure the training load continues to increase (*progressive overload*) in order to trigger neuroplasticity, while remaining safe by following strict progression rules.

**Main Principle:** Progression takes place every week (Sessions 4, 7, and 10), **ONLY IF** the subject is able to complete repetitions with an error rate or motor failure rate (e.g., mild loss of balance, imperfect steps) of less than 30%.^1^

A. **Equipment Specifications**

Based on clinical evidence of the effectiveness of *stepping* training in stroke patients, the following height standards are set:

1. **Training Block (*Step Box*):** Height **10 cm** (low), **12 cm** (standard), and **15 cm** (high).
2. **Rehabilitation Stairs:** Step height **10 cm**, **15 cm**, and **17 cm** (maximum clinical standard).

B. **6-Station TOCT Progression Table (4 Weeks)**

| **Exercise Name** | **Week 1 (Sessions**  **1-3)** | **Week 2 (Sessions**  **4-6)** | **Week 3 (Sessions**  **7-9)** | **Week 4 (Sessions**  **10-12)** |
| --- | --- | --- | --- | --- |
| 1. **Standing & Reaching** | Reach for a target at shoulder height (front/side) | Diagonal target (crossing midline) & farther away. | Reach toward the floor (trunk flexion). | Stand on a *foam mat* (unstable surface). |
| 2. **Sit-to-Stand** | Standard chair (45 cm),  hands on chest | Lower chair (40 cm) hands on chest | Standard chair (45 cm) with an added soft foam pad (2 cm), hands on chest | Low chair (40 cm) with an added soft foam pad (2 cm), hands on chest |
| 3. **Ant./Post. Stepping** | Step forward, up and down onto a 10 cm high block | Step forward, up and down onto a 12 cm high block | Step forward, up and down onto a 15 cm high block | Step forward, up and down onto a 15 cm high block with an ankle weight (0.5kg) |
| 4. **Lateral Stepping** | Step sideways, up and down onto a 10 cm high block | Step sideways, up and down onto a 12 cm high block | Step sideways, up and down onto a 15 cm high block | Step sideways, quickly up and down onto a 12 cm high block in time with a metronome |
| 5. **Forward**   **Step-Up** | Go up and down a 10 cm high step (holding the handrail) | Go up and down a 15 cm high step (holding the handrail) | Go up and down a 17 cm high step (holding the handrail) | Go up and down a 17 cm high step (without holding the handrail) |
| 6. **Heel Raise** | Stand and rise onto toes (heels lifted) on flat ground while holding on for support | Stand and rise onto toes (heels lifted) on flat ground without support | Stand on a 12 cm high block with heels hanging off the edge | Stand on one leg and rise onto toes on the paretic side with minimal assistance |

C. **Specific Instructions for the Research Assistant (RA)**

### 1. Applying the 30% Error Rule

- If, out of 10 attempts stepping onto the 12 cm block, the patient trips or requires physical assistance > 3 times, then in the following week the patient REMAINS at the 12 cm level (do not progress to 15 cm).[1]

### 2. Step Height (Station 5)

- Begin with a height of **10 cm** in the first week to build the subject’s confidence. Progression to 17 cm is only given to subjects with a good Functional Ambulation Category (FAC).[1]

### 3. Block Height (Stations 3 & 4)

- Use a wooden block *or a* stable aerobic step. Make sure the entire sole of the patient’s foot is fully placed on the block to avoid ankle injury.

### 4. Safety Monitoring

- The RA must remain on the paretic side as a *spotter.*
- Evaluate the Borg Scale at every station change. If the Borg score is > 16 (Hard), provide additional rest until the pulse rate returns to a safe range.

## 5. Post-Intervention Assessment Protocol (Post-Test)

After completing all 12 sessions within 4 weeks, the research assistant schedules the final evaluation within a 24–48 hour window after the last session. This is important for capturing the chronic adaptation effect without being confounded by acute fatigue from the final training session.

1. **Repeat Clinical Assessment:** Repeat the 6MWT, 10MWT, SWOC, and Brunnstrom Stage tests using exactly the same procedures and equipment as at baseline.^1^
2. **Cognitive Evaluation:** Repeat the MoCA-INA assessment to determine whether there is a transfer effect from dual-task training on global cognitive function.^5^
3. **Post-Test Blood Draw:** Draw blood for the BDNF biomarker. Ensure the collection time is 48 hours from the last training session and is performed between 08:00-10:00 on the day of collection.
4. **Data Reconciliation:** Check the completeness of all data in the CRF. Ensure no field is left blank without a valid explanation.^22^

## 6. Data Management, Documentation, and Quality Assurance

The integrity of the study’s results depends heavily on how research assistants manage raw data into information ready for analysis. The entire documentation process must follow strict international standards.^22^

### Application of the ALCOA+ Principles

### Every research assistant must apply the ALCOA+ principles in every data entry on the *Case Report Form* (CRF) as well as the daily *logbook*).^22^

| **ALCOA+ Element** | **Implementation in the Protocol** |
| --- | --- |
| **Attributable** | Every data entry must include the examiner’s initials and the date.^22^ |
| **Legible** | Handwriting must be clear and legible to others or to an auditor.^22^ |
| **Contemporaneous** | Data are recorded at the time the assessment takes place, not delayed until the end of the day.^22^ |
| **Original** | Data are recorded directly on the original form. Do not use scratch paper that is later copied over.^22^ |
| **Accurate** | Recorded values must match the original measurement results without manipulation.^22^ |
| **Complete & Consistent** | All fields on the form must be completed. Use consistent units of measurement.^22^ |

### Completing the Research Assistant’s Daily Logbook

*Logbook* serves as physical evidence of daily research activity. Research assistants must record:

- Arrival and departure times.^29^
- The name of the subject undergoing training and the session number.^30^
- Details of the activities performed (e.g., "Conducted the 10MWT on subject 005").^30^
- Technical issues or subject complaints during the session.^31^
- Periodic signature of the supervisor or principal investigator for validation.^30^

### Data Security and Confidentiality

In accordance with the medical code of ethics, all subject data must be anonymized using a unique code (e.g., S001, S002). Physical files (CRF) are stored in a locked cabinet, while digital data must be stored on a password-protected computer accessible only to the core research team.^7^ Data are retained for a minimum of 5–10 years in accordance with national regulations, for the purposes of future audits or CUKB (GCP) inspections.^19^

The results of this study are expected to make a real contribution to the advancement of neuroplasticity-based neuromuscular rehabilitation science in Indonesia. By demonstrating the relationship between integrated motor-cognitive training and changes in molecular biomarkers, this protocol may become a new standard in restoring adaptive walking ability for subacute stroke patients, enabling them to function independently in the community once again.^1^

All research assistants are expected to carry out this protocol with full dedication, precision, and high scientific integrity, in order to achieve reputable international publication standards and, most importantly, to improve the quality of life of stroke survivors.^1^

#### References

1. RDDU non final.pdf
2. Differential Effects of Cognitive vs. Motor Dual-Task Training in Stroke Rehabilitation: A Precision-Focused Meta-Analysis | medRxiv, accessed March 29, 2026, [https://www.medrxiv.org/content/10.64898/2026.01.23.26344517v1.full-text](https://www.google.com/url?q=https://www.medrxiv.org/content/10.64898/2026.01.23.26344517v1.full-text&sa=D&source=editors&ust=1774754033760128&usg=AOvVaw2GSlVYCqDhxBj9Cwkq7hwQ)
3. Dual Task Effects on Speed and Accuracy During Cognitive and Upper Limb Motor Tasks in Adults With Stroke Hemiparesis - PMC, accessed March 29, 2026, [https://pmc.ncbi.nlm.nih.gov/articles/PMC8250862/](https://www.google.com/url?q=https://pmc.ncbi.nlm.nih.gov/articles/PMC8250862/&sa=D&source=editors&ust=1774754033761107&usg=AOvVaw2OJp_vBK6pZVhuOjpUk8qv)
4. Upper Extremity-Cognitive Dual-Task Capacity Post-Stroke - PMC - NIH, accessed March 29, 2026, [https://pmc.ncbi.nlm.nih.gov/articles/PMC12065953/](https://www.google.com/url?q=https://pmc.ncbi.nlm.nih.gov/articles/PMC12065953/&sa=D&source=editors&ust=1774754033761852&usg=AOvVaw2g82Qwn-JDTATmGBlS9F32)
5. A meta-analysis of the effects of dual-task training on cognitive function in stroke patients, accessed March 29, 2026, [https://www.frontiersin.org/journals/neurology/articles/10.3389/fneur.2025.1417364/full](https://www.google.com/url?q=https://www.frontiersin.org/journals/neurology/articles/10.3389/fneur.2025.1417364/full&sa=D&source=editors&ust=1774754033762848&usg=AOvVaw3zRinpT1rE7Poqh8rJBlNJ)
6. Comparing the effectiveness of dual-task and single-task training on walking function in stroke recovery: A systematic review and meta-analysis - PMC, accessed March 29, 2026, [https://pmc.ncbi.nlm.nih.gov/articles/PMC11902943/](https://www.google.com/url?q=https://pmc.ncbi.nlm.nih.gov/articles/PMC11902943/&sa=D&source=editors&ust=1774754033763848&usg=AOvVaw0BbpIVdpoRwhXCbcDWZ1RG)
7. Standard Operating Procedure (SOP) - National Institute on Drug Abuse, accessed March 29, 2026, [https://nida.nih.gov/sites/default/files/nida_gcp_training_sop.pdf](https://www.google.com/url?q=https://nida.nih.gov/sites/default/files/nida_gcp_training_sop.pdf&sa=D&source=editors&ust=1774754033764659&usg=AOvVaw0ZriELHSY76oKJ2dEsEv67)
8. Pedoman Cara Uji Klinik yang Baik (CUKB) di Indonesia, accessed March 29, 2026, [https://id.gmp-platform.com/app/uploads/2020/08/Pedoman-Cara-Uji-Klinik-Yang-Baik-Di-Indonesia.pdf](https://www.google.com/url?q=https://id.gmp-platform.com/app/uploads/2020/08/Pedoman-Cara-Uji-Klinik-Yang-Baik-Di-Indonesia.pdf&sa=D&source=editors&ust=1774754033765525&usg=AOvVaw2mP6RfRQ9mUJMoq9CSzN-y)
9. PERATURAN KEPALA BADAN PENGAWAS OBAT DAN MAKANAN REPUBLIK INDONESIA NOMOR 9 TAHUN 2014 TENTANG TATA LAKSANA PERSETUJUAN UJI KLIN - KEP UPNVJ, accessed March 29, 2026, [https://kep.upnvj.ac.id/assets/referensi/10.%20tata%20laksana%20persetujuan%20uji%20klinik%202014.pdf](https://www.google.com/url?q=https://kep.upnvj.ac.id/assets/referensi/10.%20tata%20laksana%20persetujuan%20uji%20klinik%202014.pdf&sa=D&source=editors&ust=1774754033766498&usg=AOvVaw3eo6PEUzhzHfD_wXke628y)
10. Good Clinical Practice Training | Grants & Funding, accessed March 29, 2026, [https://grants.nih.gov/policy-and-compliance/policy-topics/clinical-trials/good-clinical-training](https://www.google.com/url?q=https://grants.nih.gov/policy-and-compliance/policy-topics/clinical-trials/good-clinical-training&sa=D&source=editors&ust=1774754033767219&usg=AOvVaw3B8k77VdCU9xzaWDYQRk-m)
11. BADAN PENGAWAS OBAT DAN MAKANAN KEPUTUSAN KEPALA BADAN PENGAWAS OBAT DAN MAKANAN REPUBLIK INDONESIA NOMOR 02002/SK/KBPOM TENTANG - JDIH Badan POM, accessed March 29, 2026, [https://jdih.pom.go.id/download/rule/875/02002%7CSK%7CKBPOM/2001](https://www.google.com/url?q=https://jdih.pom.go.id/download/rule/875/02002%7CSK%7CKBPOM/2001&sa=D&source=editors&ust=1774754033767806&usg=AOvVaw0Qh1vyGG4NPSKNmFs0qFZf)
12. KODE ETIK KEDOKTERAN INDONESIA DAN PEDOMAN PELAKSANAAN KODE ETIK KEDOKTERAN INDONESIA, accessed March 29, 2026, [https://luk.staff.ugm.ac.id/atur/sehat/Kode-Etik-Kedokteran.pdf](https://www.google.com/url?q=https://luk.staff.ugm.ac.id/atur/sehat/Kode-Etik-Kedokteran.pdf&sa=D&source=editors&ust=1774754033768320&usg=AOvVaw3NaDHZmSoGy5PMukDP7Wa6)
13. Cara menjadi Asisten Peneliti - Keterampilan & Deskripsi Pekerjaan – Jobstreet, accessed March 29, 2026, [https://id.jobstreet.com/id/career-advice/role/research-assistant](https://www.google.com/url?q=https://id.jobstreet.com/id/career-advice/role/research-assistant&sa=D&source=editors&ust=1774754033768903&usg=AOvVaw2iiuS2zEt76Rw0hswCwbmJ)
14. pedoman akademik program studi dokter spesialis neurologi - FK UNPAD, accessed March 29, 2026, [https://fk.unpad.ac.id/wp-content/uploads/2024/05/Pedoman-Akademik-Neurologi.pdf](https://www.google.com/url?q=https://fk.unpad.ac.id/wp-content/uploads/2024/05/Pedoman-Akademik-Neurologi.pdf&sa=D&source=editors&ust=1774754033769486&usg=AOvVaw1zWbuE0k_DmSmjwzese-Jp)
15. Kode Etik Kedokteran Indonesia - Equals.id, accessed March 29, 2026, [https://equalsid.or.id/dokumen/101124182354_KODEKI-Tahun-2012.pdf%20%C2%B7%20versi%201.pdf](https://www.google.com/url?q=https://equalsid.or.id/dokumen/101124182354_KODEKI-Tahun-2012.pdf%20%C2%B7%20versi%201.pdf&sa=D&source=editors&ust=1774754033769988&usg=AOvVaw1bMIljPcArZH_l2pHgDL-b)
16. Clinical Research SOPs - Research Quality and Compliance - The University of Utah, accessed March 29, 2026, [https://qualitycompliance.research.utah.edu/sop-library/](https://www.google.com/url?q=https://qualitycompliance.research.utah.edu/sop-library/&sa=D&source=editors&ust=1774754033770460&usg=AOvVaw1kj8LFOMTH5thpjNDAXHvG)
17. SOP #104 - Conduct of Clinical Study - Kent State University, accessed March 29, 2026, [https://www-s3-live.kent.edu/s3fs-root/s3fs-public/file/SOP_104_ClinicalStudyConduct.docx?VersionId=jnO4I1JG8vZ4ZMmSTXK5RaYBGOc57sEV](https://www.google.com/url?q=https://www-s3-live.kent.edu/s3fs-root/s3fs-public/file/SOP_104_ClinicalStudyConduct.docx?VersionId=jnO4I1JG8vZ4ZMmSTXK5RaYBGOc57sEV&sa=D&source=editors&ust=1774754033771148&usg=AOvVaw0LYuhgOAuOWHKthyd-t-JQ)
18. SOPs for Clinical Research | Institute for Global Health and Infectious Diseases, accessed March 29, 2026, [https://globalhealth.unc.edu/research/ighid-research-management/sops-for-clinical-research/](https://www.google.com/url?q=https://globalhealth.unc.edu/research/ighid-research-management/sops-for-clinical-research/&sa=D&source=editors&ust=1774754033771788&usg=AOvVaw1Qi_fOCR3nP-89QOssBKUp)
19. Standard Operating Procedures (SOPs) in Clinical Trials | Credevo Articles, accessed March 29, 2026, [https://credevo.com/articles/2023/01/05/standard-operating-procedures-sops-in-clinical-trials/](https://www.google.com/url?q=https://credevo.com/articles/2023/01/05/standard-operating-procedures-sops-in-clinical-trials/&sa=D&source=editors&ust=1774754033772413&usg=AOvVaw3w9ePiM3Udc1FGSOQPX3da)
20. KEPUTUSAN MENTERI KESEHATAN REPUBLIK INDONESIA NOMOR HK.01.07/MENKES/1458/2023 TENTANG PENYELENGGARAAN PENELITIAN KLINIK DI RU, accessed March 29, 2026, [https://keslan.kemkes.go.id/unduhan/fileunduhan_1690693655_545916.pdf](https://www.google.com/url?q=https://keslan.kemkes.go.id/unduhan/fileunduhan_1690693655_545916.pdf&sa=D&source=editors&ust=1774754033773190&usg=AOvVaw0Do-EUPfKTmxP8W0lBJYIz)
21. PROSEDUR OPERASIONAL BAKU (SOP) - SIMKEPKES - Universitas Hasanuddin, accessed March 29, 2026, [https://simkepkes.med.unhas.ac.id/assets/uploads/media/02a56e16eef6473c6300aaf1cd8735b7.pdf](https://www.google.com/url?q=https://simkepkes.med.unhas.ac.id/assets/uploads/media/02a56e16eef6473c6300aaf1cd8735b7.pdf&sa=D&source=editors&ust=1774754033774064&usg=AOvVaw1oOJetiXM-m0hacdbhXCtH)
22. GCP Guidelines Mastery: Essential Practices for Clinical Trials - CCRPS, accessed March 29, 2026, [https://ccrps.org/clinical-research-blog/gcp-guidelines-mastery-essential-practices-for-clinical-trials](https://www.google.com/url?q=https://ccrps.org/clinical-research-blog/gcp-guidelines-mastery-essential-practices-for-clinical-trials&sa=D&source=editors&ust=1774754033774804&usg=AOvVaw22YvK8GrRohFTlcY8CThfS)
23. ct-310 clinical research data management - University of South Alabama, accessed March 29, 2026, [https://www.southalabama.edu/colleges/com/research/resources/ct310clinicaldatamanagement.pdf](https://www.google.com/url?q=https://www.southalabama.edu/colleges/com/research/resources/ct310clinicaldatamanagement.pdf&sa=D&source=editors&ust=1774754033775545&usg=AOvVaw1aAL7-ipEl51_WUp3eUA1V)
24. Effective Data Collection & Management for Research Assistants - CCRPS, accessed March 29, 2026, [https://ccrps.org/clinical-research-blog/effective-data-collection-amp-management-for-research-assistants](https://www.google.com/url?q=https://ccrps.org/clinical-research-blog/effective-data-collection-amp-management-for-research-assistants&sa=D&source=editors&ust=1774754033776276&usg=AOvVaw1VxYYGTlpkcwh-xZoirXqB)
25. Pedoman dan Standar Etik Penelitian dan Pengembangan Kesehatan Nasional - KEPKN, accessed March 29, 2026, [https://kepkn.kemkes.go.id/assets/img/keppkn/file/BUKU_PEDOMAN_DAN_STANDAR_ETIK_PENELITIAN_DAN_PENGEMBANGAN_KESEHATAN_NASIONAL(KEPKN).pdf](https://www.google.com/url?q=https://kepkn.kemkes.go.id/assets/img/keppkn/file/BUKU_PEDOMAN_DAN_STANDAR_ETIK_PENELITIAN_DAN_PENGEMBANGAN_KESEHATAN_NASIONAL(KEPKN).pdf&sa=D&source=editors&ust=1774754033777150&usg=AOvVaw3jSFSiYB4JJImWFfWFW2YX)
26. Standard Operating Procedures for Clinical Research at USF Health CRC QUALITY CONTROL RESPONSIBILITY, accessed March 29, 2026, [https://health.usf.edu/-/media/v3/usf-health/medicine/Research/OCR/Standard-Operating-Procedures/SOP_601_QualityControl.ashx](https://www.google.com/url?q=https://health.usf.edu/-/media/v3/usf-health/medicine/Research/OCR/Standard-Operating-Procedures/SOP_601_QualityControl.ashx&sa=D&source=editors&ust=1774754033778164&usg=AOvVaw3G6VErLXqPyvZUez18Jb5R)
27. PROSEDUR OPERASIONAL BAKU (POB) - E-Komisi Etik Penelitian Universitas Padjadjaran, accessed March 29, 2026, [https://admkep.unpad.ac.id/sop/POB_2022.pdf](https://www.google.com/url?q=https://admkep.unpad.ac.id/sop/POB_2022.pdf&sa=D&source=editors&ust=1774754033778838&usg=AOvVaw2A01vWhHCZgrIcfhIb5xSs)
28. SOPs BIRAC CTN Clinical Research Secretariat - National Cancer Grid, accessed March 29, 2026, [https://www.ncgindia.org/assets/ncg-research/sops/sops-birac-ctn-clinical-research-secretariat.pdf](https://www.google.com/url?q=https://www.ncgindia.org/assets/ncg-research/sops/sops-birac-ctn-clinical-research-secretariat.pdf&sa=D&source=editors&ust=1774754033779663&usg=AOvVaw3_VB1wyJuF-JjVX5sDs-JQ)
29. LOGBOOK - Bagian IKM-KP FK UNS, accessed March 29, 2026, [http://ikm.fk.uns.ac.id/wp-content/uploads/2015/10/Log-Book.doc](https://www.google.com/url?q=http://ikm.fk.uns.ac.id/wp-content/uploads/2015/10/Log-Book.doc&sa=D&source=editors&ust=1774754033780335&usg=AOvVaw0Fm47OGq6YhmG0GAhs3wAP)
30. Log Book Penelitian Kebidanan D-IV | PDF - Scribd, accessed March 29, 2026, [https://id.scribd.com/document/511081200/log-book](https://www.google.com/url?q=https://id.scribd.com/document/511081200/log-book&sa=D&source=editors&ust=1774754033780887&usg=AOvVaw2N4SqR241tegLoFTiHmGh9)
31. Contoh Logbook Penelitian Skripsi | PDF - Scribd, accessed March 29, 2026, [https://www.scribd.com/document/453815779/LOG-BOOK-PENELITIAN-docx](https://www.google.com/url?q=https://www.scribd.com/document/453815779/LOG-BOOK-PENELITIAN-docx&sa=D&source=editors&ust=1774754033781430&usg=AOvVaw1cVwXU-7RCR6gfQhFGoQPW)
32. 2.b Log Book Penelitian | PDF - Scribd, accessed March 29, 2026, [https://id.scribd.com/document/495364996/2-b-Log-Book-Penelitian](https://www.google.com/url?q=https://id.scribd.com/document/495364996/2-b-Log-Book-Penelitian&sa=D&source=editors&ust=1774754033781921&usg=AOvVaw3q-5Zk8I082SOKPw7u8Qxb)
33. Good Clinical Practice Research Guidelines Reviewed, Emphasis Given to Responsibilities of Investigators: Second Article in a Series - PMC, accessed March 29, 2026, [https://pmc.ncbi.nlm.nih.gov/articles/PMC2794015/](https://www.google.com/url?q=https://pmc.ncbi.nlm.nih.gov/articles/PMC2794015/&sa=D&source=editors&ust=1774754033782561&usg=AOvVaw2ynWfV0D-Cd1cHcCqsMKkz)
