# Supplementary material for: The effect of dual task-oriented circuit training on neuroplasticity and walking adaptability in subacute stroke patients: A pilot and feasibility study
Source: PLoS One. 2026 Oct 27;21(10):e0361255. doi: 10.1371/journal.pone.0361255 (PMC13712583; doi:10.1371/journal.pone.0361255)
Supplement: Supplementary file 4 [file pone.0361255.s004.docx]

**Protokol Penanganan KTD**

**Protokol Kejadian Tak Diinginkan (KTD)**

**Saat Latihan Terapi Berjalan**

A. **Tujuan**

Protokol ini dibuat dengan tujuan untuk menjamin keselamatan pasien dengan melakukan deteksi dini, penanganan cepat, dan rujukan tepat pada kejadian tidak diinginakn (KTD) meliputi jatuh, sesak nafas berat, nyeri dada, sinkop maupun presinkop, dan cedera otot saat latihan.

B. **Tanda Bahaya (Red Flags)**

Latihan harus segera dihentikan apabila muncul:

1. Respirasi
   a) SpO₂ < 92%
   b) Sesak berat / tidak bisa bicara
   c) Penggunaan otot bantu napas berlebihan
2. Kardiovaskular
a) Denyut jantung 50-70% HR Maximal
b) Tekanan darah > 180/100 mmHg atau 90/60 mmHg
c) Pusing / hampir pingsan
d) Nyeri dada seperti angina
3. Lainnya
   a) Kramp otot hebat
   b) Pucat / keringat dingin
   c) Kehilangan keseimbangan
C. **Prosedur Umum**
1. STOP latihan segera
2. Posisikan pasien duduk / semi-Fowler atau supine bila sinkop
3. Monitoring cepat dengan menilai nadi, tekanan darah, SpO2, dan RR
4. Tenangkan pasien
5. Aktifkan bantuan (Dokter/Perawat/Terapis)
6. Dokumentasikan kejadian
D. **Protokol Spesifik**
1. Jika Pasien Jatuh
a) Cek kesadaran pasien (GCS), nyeri, deformitas
b) Imobilisasi jika dicurigai cedera, pindahkan dengan teknik aman
c) Laporkan pada Dokter dengan memberikan hasil observasi vital sign
d) Dokumentasi kejadian
2. Jika Sesak Napas Berat
a) Hentikan latihan
b) Posisikan pasien tripod / duduk condong ke depan dan mengajarkan atur napas
c) Berikan oksigen dan lakukan monitoring SpO₂ , RR, serta tanda distress
d) Lakukan rujuk ke Unit Gawat Darurat (UGD) Rumah Sakit
3. Jika Nyeri Dada
a) Hentikan latihan
b) Posisikan pasien duduk / semi-fowler
c) Evaluasi: lokasi nyeri, durasi, karakteristik nyeri
d) Monitoring HR, TD, SpO₂ , RR, dan SpO₂ dan segera hubungi Dokter
e) Apabila nyeri > 15 menit, menjalar ke lengan/rahang disertai sesak / keringat dingin (khas angina)
f) Lakukan rujuk ke Unit Gawat Darurat (UGD) Rumah Sakit
4. Jika Sinkop / Presinkop
   a) Hentikan latihan
   b) Posisikan pasien supine serta elevasi kaki
   c) Pastikan jalan nafas aman dan cek respon pasien
g) Berikan oksigen dan lakukan monitoring SpO₂ , RR, TD, HR
h) Jika tidak sadar, tidak ada napas, tidak ada nadi segera aktifkan code blue / emergency system
i) Lakukan rujuk ke Unit Gawat Darurat (UGD) Rumah Sakit
E. **Kriteria Rujukan Darurat**

Segera rujuk jika:

a) SpO₂ < 88 % persisten
b) Nyeri dada tidak hilang
c) Penurunan kesadaran
d) Aritmia
e) Hipotensi
f) Sinkop
F. **Pencegahan**
1. Sebelum latihan:
a) Cek vital sign
b) Cek SpO₂ baseline
c) Pastikan pasien stabil
d) Gunakan Borg scale
e) Edukasi tanda bahaya
2. Saat latihan:
   a) Gunakan interval training
   b) Monitoring kontinu
   c) Gunakan alat bantu bila perlu
G. **Dokumentasi**

Catat :

1. Waktu kejadian
2. Jenis KTD
3. Tanda vital
4. Tindakan yang dilakukan
5. Respons pasien
6. Rujukan

**Standar Operasional Prosedur KTD**

**Saat Latihan Terapi Berjalan**

**A. Identitas SOP**

| Komponen | Isi |
| --- | --- |
| Judul | Penatalaksanaan Kejadian Tidak Diinginkan (KTD) Saat Latihan Berjalan |
| Unit | Rehabilitasi Medik |
| Sasaran | Pasien rawat jalan |
| Pelaksana | Fisioterapis, Dokter Rehabilitasi Medik, Perawat |
| Tujuan | Menjamin keselamatan pasien saat terapi latihan |

**B. Prosedur Penanganan KTD**

| **No** | **Kondisi** | **Tindakan Utama** | **Monitoring** | **Tindak Lanjut** |
| --- | --- | --- | --- | --- |
| 1 | Semua KTD | Hentikan latihan, amankan pasien | HR, BP, SpO₂ | Panggil bantuan |
| 2 | Jatuh | Jangan langsung bangun, cek cedera | GCS, nyeri | Rujuk dokter |
| 3 | Dizziness / pusing | Dudukkan pasien, elevasi kaki bila perlu | BP, HR | Evaluasi ortostatik |
| 4 | Hipertensi | Hentikan latihan, istirahat | BP | Jika >180/110 → rujuk UGD |
| 5 | Fatigue berat | Istirahat, pacing | Borg scale | Modifikasi latihan |
| 6 | Sesak napas berat | Posisi tripod, breathing control, O₂ bila perlu | SpO₂, RR | Jika tidak membaik → rujuk UGD |
| 7 | Nyeri dada | Hentikan latihan, posisikan duduk, evaluasi nyeri | HR, BP, SpO2 | Rujuk segera |
| 9 | Desaturasi (<88%) | Hentikan latihan, pasang oksigen, istirahat | Evaluasi ulang program | SpO2 target >90% |

C. **Kriteria Hentikan Latihan**

| Parameter | Batas |
| --- | --- |
| SpO₂ | < 88% |
| Borg dyspnea | ≥ 15 |
| HR | Tidak stabil / aritmia |
| BP | Tekanan darah > 180/100 mmHg atau 90/60 mmHg |
| Gejala | Nyeri dada, pusing, sesak napas, sinkop/presinkop |

D. **Dokumentasi**

| Waktu kejadian |  |
| --- | --- |
| Jenis KTD |  |
| Vital sign |  |
| Intervensi |  |
| Respons pasien |  |
| Rujukan |  |

**Diagram Alur SOP KTD**

**Saat Latihan Terapi Berjalan**

**SOP Pelaporan KTD Kejadian Tak Diinginkan (KTD)**

**Saat Latihan Terapi Berjalan**
