# Supplementary material for: The effect of dual task-oriented circuit training on neuroplasticity and walking adaptability in subacute stroke patients: A pilot and feasibility study
Source: PLoS One. 2026 Oct 27;21(10):e0361255. doi: 10.1371/journal.pone.0361255 (PMC13712583; doi:10.1371/journal.pone.0361255)
Supplement: Supplementary file 5 [file pone.0361255.s005.docx]

**Adverse Event (AE) Management Protocol**

**Adverse Event (AE) Protocol**

**During Walking Therapy Exercise**

A. **Purpose**

This protocol was developed to ensure patient safety through early detection, rapid management, and appropriate referral for adverse events (AE), including falls, severe shortness of breath, chest pain, syncope or presyncope, and muscle injury during exercise.

B. **Warning Signs (Red Flags)**

Exercise must immediately be stopped if any of the following occur:

1. Respiratory
   a) SpO₂ < 92%
   b) Severe shortness of breath / unable to speak
   c) Excessive use of accessory respiratory muscles
2. Cardiovascular
a) Heart rate 50-70% HR Maximum
b) Blood pressure > 180/100 mmHg or 90/60 mmHg
c) Dizziness / near fainting
d) Chest pain resembling angina
3. Other
   a) Severe muscle cramps
   b) Pallor / cold sweat
   c) Loss of balance
C. **General Procedure**
1. STOP exercise immediately
2. Position the patient sitting / semi-Fowler, or supine if syncope occurs
3. Perform rapid monitoring by assessing pulse, blood pressure, SpO2, and RR
4. Reassure the patient
5. Activate assistance (Doctor/Nurse/Therapist)
6. Document the event
D. **Specific Protocols**
1. If the Patient Falls
a) Check patient consciousness (GCS), pain, deformity
b) Immobilize if injury is suspected, move using a safe technique
c) Report to the Doctor with the vital sign observation results
d) Document the event
2. If Severe Shortness of Breath
a) Stop exercise
b) Position the patient in tripod position / sitting leaning forward, and teach breathing control
c) Give oxygen and monitor SpO₂, RR, and signs of distress
d) Refer to the Hospital Emergency Department (ED)
3. If Chest Pain
a) Stop exercise
b) Position the patient sitting / semi-fowler
c) Evaluate: pain location, duration, characteristics of the pain
d) Monitor HR, BP, SpO₂, RR, and SpO₂, and immediately contact the Doctor
e) If pain > 15 minutes, radiating to the arm/jaw accompanied by shortness of breath / cold sweat (typical of angina)
f) Refer to the Hospital Emergency Department (ED)
4. If Syncope / Presyncope
   a) Stop exercise
   b) Position the patient supine and elevate the legs
   c) Ensure the airway is secure and check patient responsiveness
g) Give oxygen and monitor SpO₂, RR, BP, HR
h) If unresponsive, not breathing, no pulse immediately activate the code blue / emergency system
i) Refer to the Hospital Emergency Department (ED)
E. **Emergency Referral Criteria**

Refer immediately if:

a) SpO₂ < 88 % persistently
b) Chest pain does not resolve
c) Decreased consciousness
d) Arrhythmia
e) Hypotension
f) Syncope
F. **Prevention**
1. Before exercise:
a) Check vital signs
b) Check baseline SpO₂
c) Ensure the patient is stable
d) Use the Borg scale
e) Educate on warning signs
2. During exercise:
   a) Use interval training
   b) Continuous monitoring
   c) Use assistive devices if needed
G. **Documentation**

Record:

1. Time of event
2. Type of AE
3. Vital signs
4. Actions taken
5. Patient response
6. Referral

**Standard Operating Procedure for AE**

**During Walking Therapy Exercise**

**A. SOP Identity**

| Component | Content |
| --- | --- |
| Title | Management of Adverse Events (AE) During Walking Exercise |
| Unit | Medical Rehabilitation |
| Target | Outpatients |
| Implementers | Physiotherapists, Rehabilitation Medicine Physicians, Nurses |
| Purpose | Ensure patient safety during exercise therapy |

**B. AE Management Procedure**

| **No** | **Condition** | **Primary Action** | **Monitoring** | **Follow-up** |
| --- | --- | --- | --- | --- |
| 1 | All AE | Stop exercise, secure the patient | HR, BP, SpO₂ | Call for assistance |
| 2 | Fall | Do not stand up immediately, check for injury | GCS, pain | Refer to doctor |
| 3 | Dizziness | Sit the patient down, elevate legs if needed | BP, HR | Orthostatic evaluation |
| 4 | Hypertension | Stop exercise, rest | BP | If >180/110 → refer to ED |
| 5 | Severe fatigue | Rest, pacing | Borg scale | Modify exercise |
| 6 | Severe shortness of breath | Tripod position, breathing control, O₂ if needed | SpO₂, RR | If not improving → refer to ED |
| 7 | Chest pain | Stop exercise, position sitting, evaluate pain | HR, BP, SpO2 | Refer immediately |
| 9 | Desaturation (<88%) | Stop exercise, administer oxygen, rest | Re-evaluate program | SpO2 target >90% |

C. **Exercise Stopping Criteria**

| Parameter | Threshold |
| --- | --- |
| SpO₂ | < 88% |
| Borg dyspnea | ≥ 15 |
| HR | Unstable / arrhythmia |
| BP | Blood pressure > 180/100 mmHg or 90/60 mmHg |
| Symptoms | Chest pain, dizziness, shortness of breath, syncope/presyncope |

D. **Documentation**

| Time of event |  |
| --- | --- |
| Type of AE |  |
| Vital signs |  |
| Intervention |  |
| Patient response |  |
| Referral |  |

**AE SOP Flow Diagram**

**During Walking Therapy Exercise**

**SOP for Reporting AE (Adverse Events)**

**During Walking Therapy Exercise**
